# Supplementary material for: ImmGen report: sexual dimorphism in the immune system transcriptome
Source: Nat Commun. 2019 Sep 20;10:4295. doi: 10.1038/s41467-019-12348-6 (PMC6754408; doi:10.1038/s41467-019-12348-6)
Supplement: Supplementary file 1 — Supplementary Information [file 41467_2019_12348_MOESM1_ESM.pdf]

Supplementary Information

ImmGen Report: Sexual Dimorphism in the Immune System Transcriptome

Gal-Oz et al.

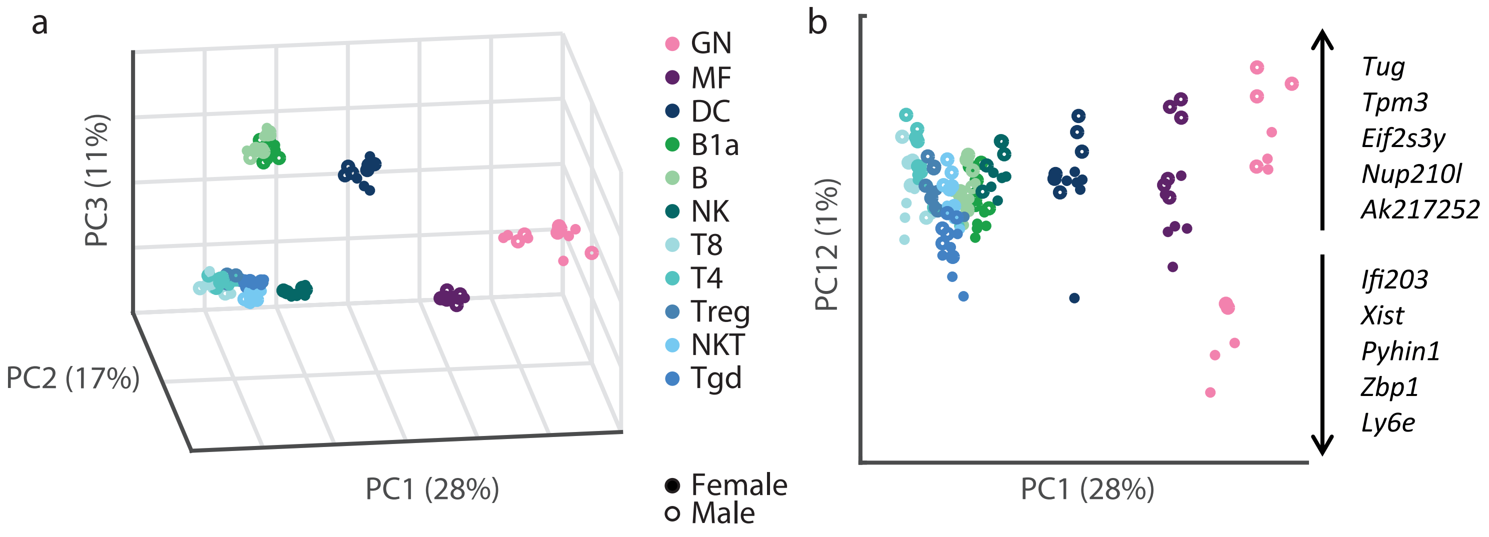

Supplementary Figure 1: Overall effect of immune cell type and sex by Principal component analysis (PCA). a, Components 1-3, explaining ~55.6% of the variance in the data. Male (empty circles) and female (filled circles) samples from datasets A and B are shown, based on the expression of 2904 genes that are expressed in both datasets above the noise threshold (see Methods). Cell types are marked by colors. b, The first versus the 12th principal component, explaining 1.04% of the variance, separating male and female samples. Top most contributing genes to the 12th principal component are listed by direction of contribution. Source data for (a, b) are provided as a Source Data file.

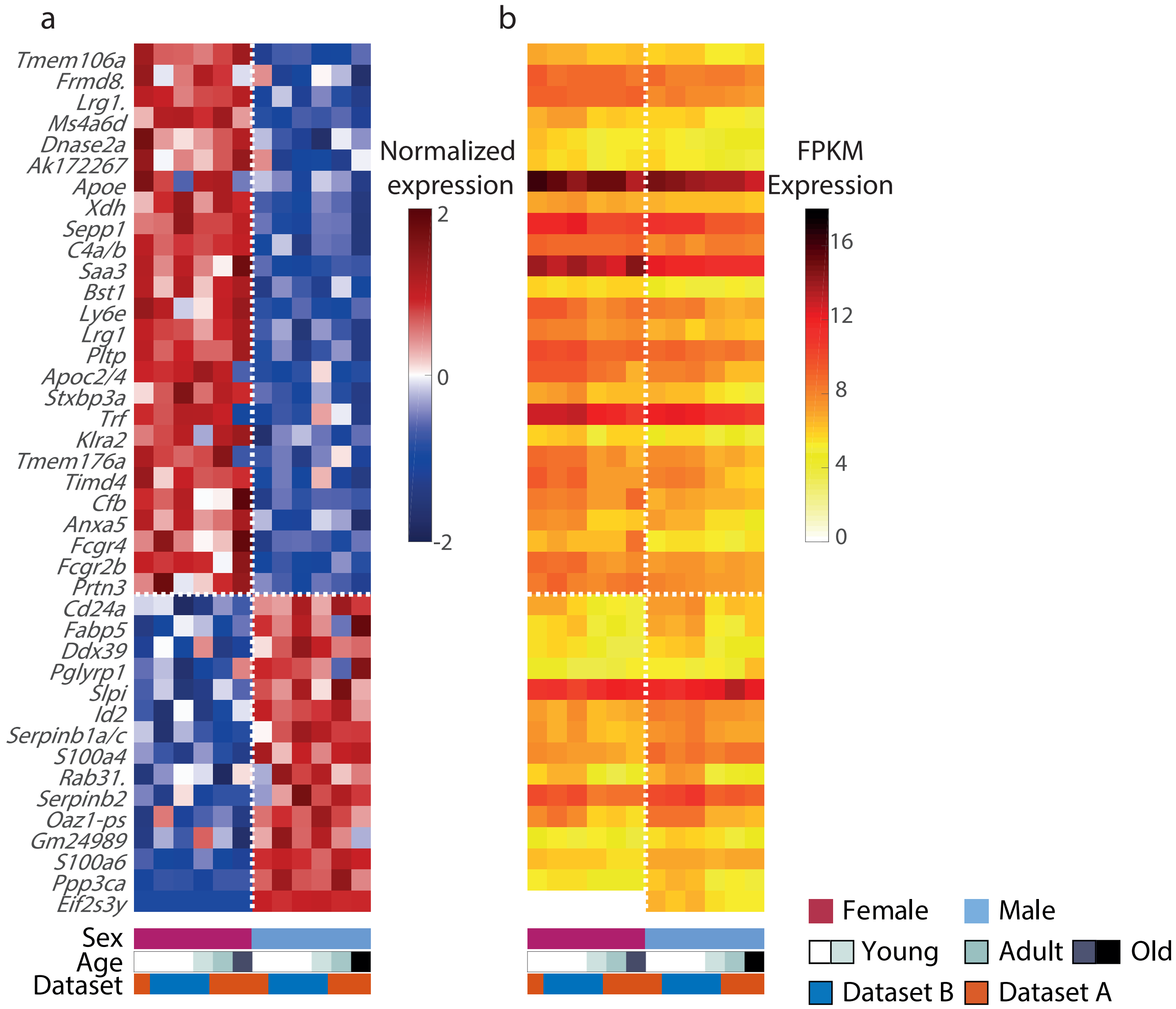

Supplementary Figure 2: Macrophage-specific sex signature. Heatmap of (a) relative expression (expression values are trimmed to range [-2,2]) and (b) FPKM levels of male and female SDEGs in macrophages from datasets A and B. Genes are sorted by female-male fold change. Colorbars indicating sex, age (6 weeks and 2,6,17/20 months) and dataset (A and B) are shown below the heatmaps. For genes with multiple names, only the first name is shown, and then period. Source data for (a, b) are provided as a Source Data file.

a

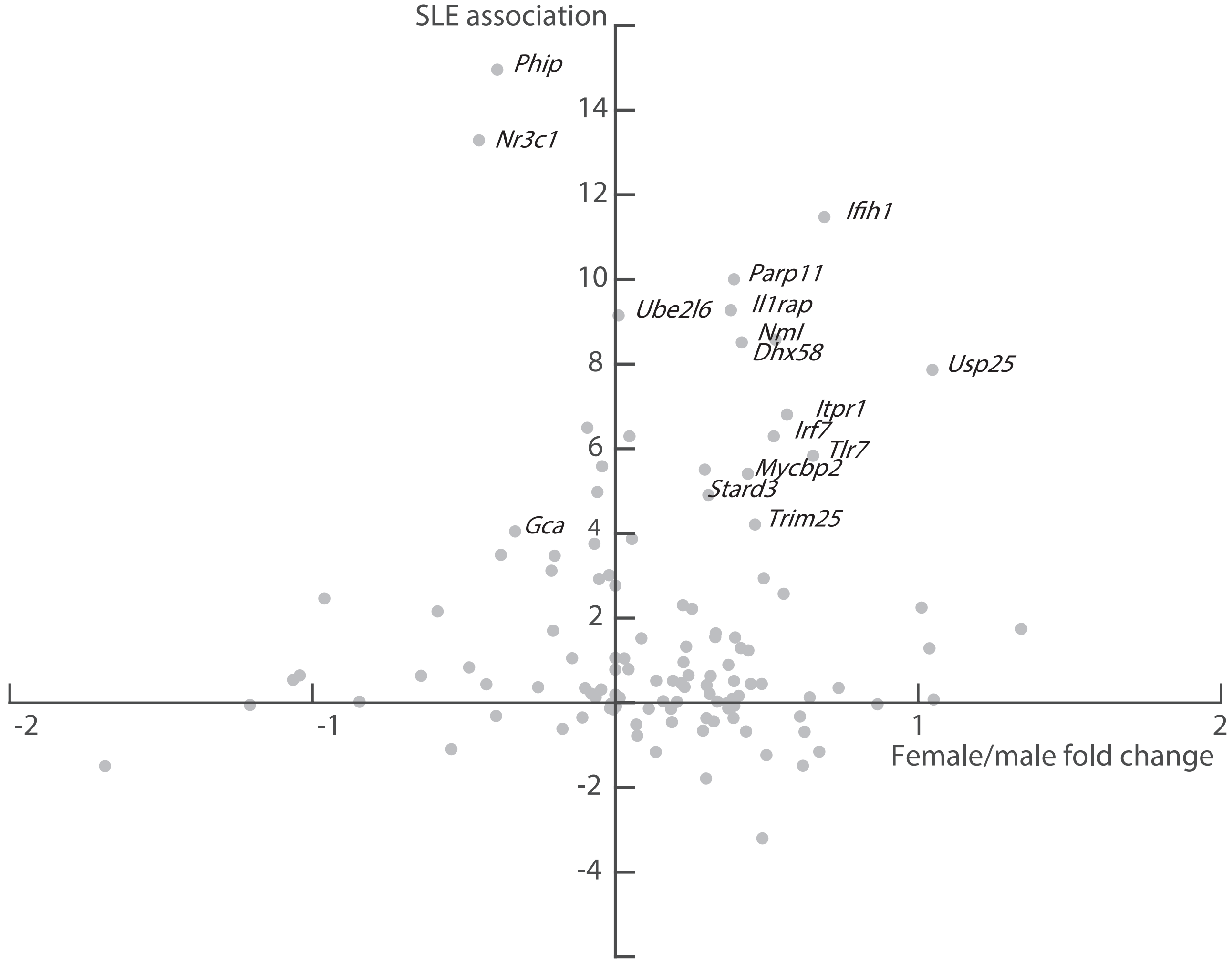

b

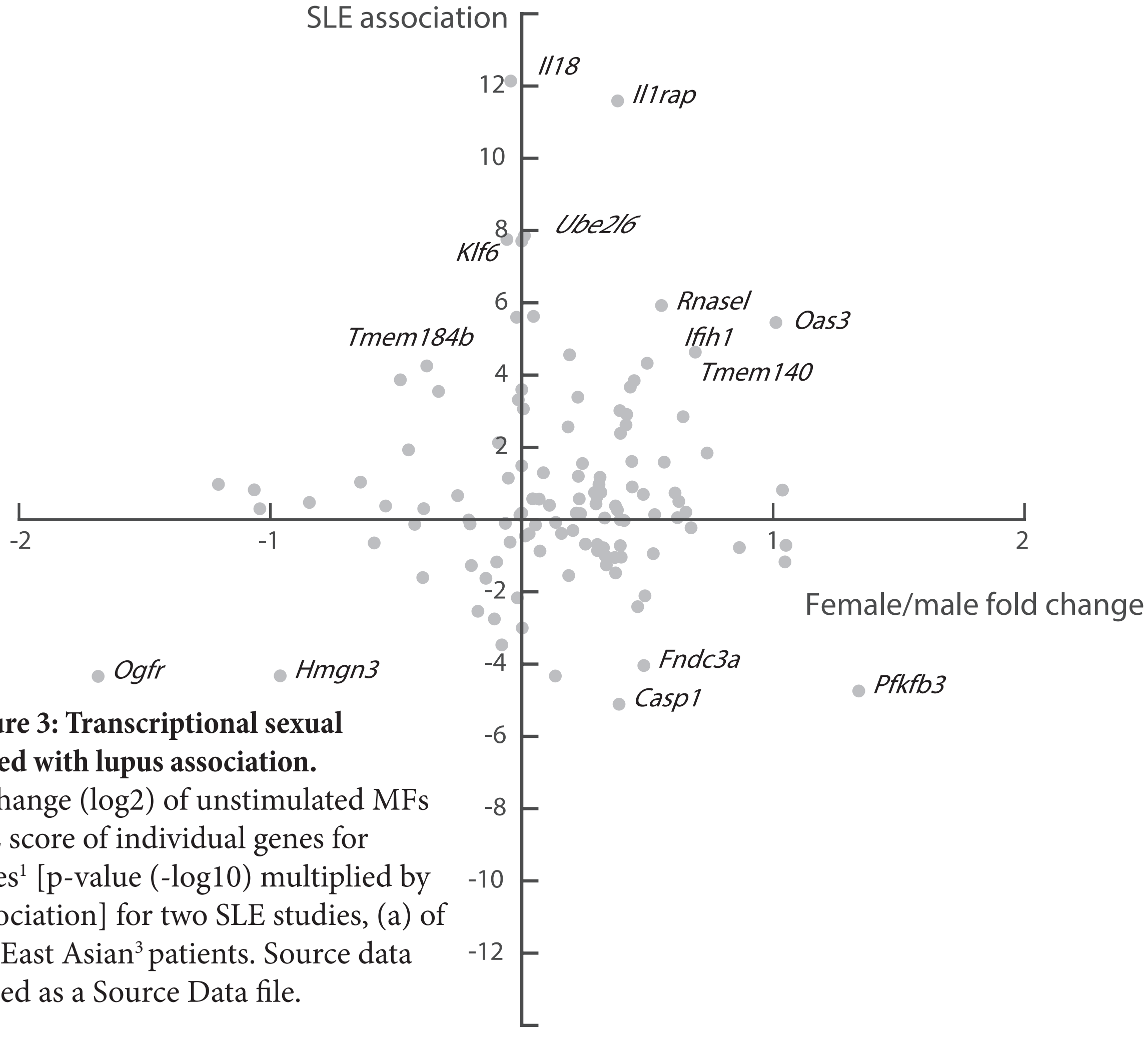

Supplementary Figure 3: Transcriptional sexual dimorphism is related with lupus association. Female-male fold change (log2) of unstimulated MFs compared with SLE score of individual genes for MF-ISGs. SLE scores[1] [p-value (-log10) multiplied by the direction of association] for two SLE studies, (a) of European[2], and (b) East Asian[3] patients. Source data for (a, b) are provided as a Source Data file.

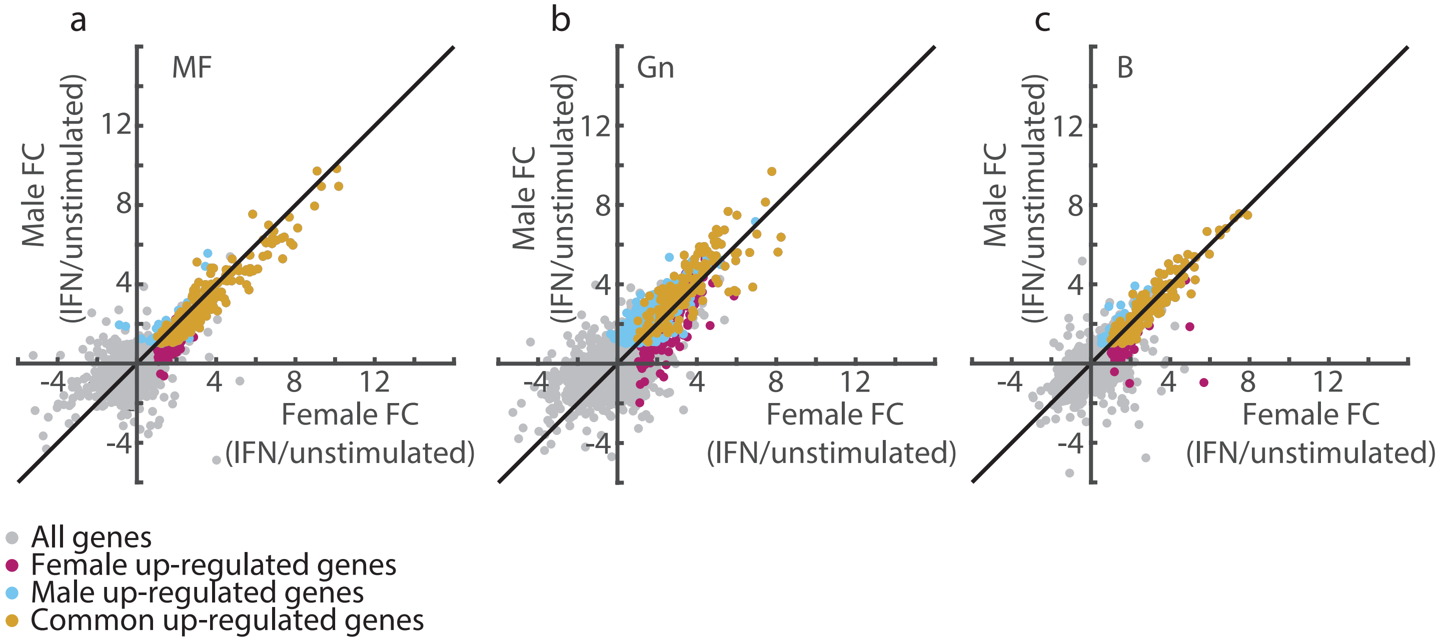

Supplementary Figure 4: Male and female responses to IFN stimulation are similar. Shown are scatter plots of the differences between male–female mean fold change of expression (3 replicates, dataset B) in (a) macrophages, (b) granulocytes and (c) B cells. Each dot represents a gene. Genes that are induced only in females, only in males, or in both sexes are colored pink, blue, and gold, respectively. Induction criteria are t-test pFDR < 0.05 and IFN-unstimulated fold change>2. Source data for (a-c) are provided as a Source Data file.

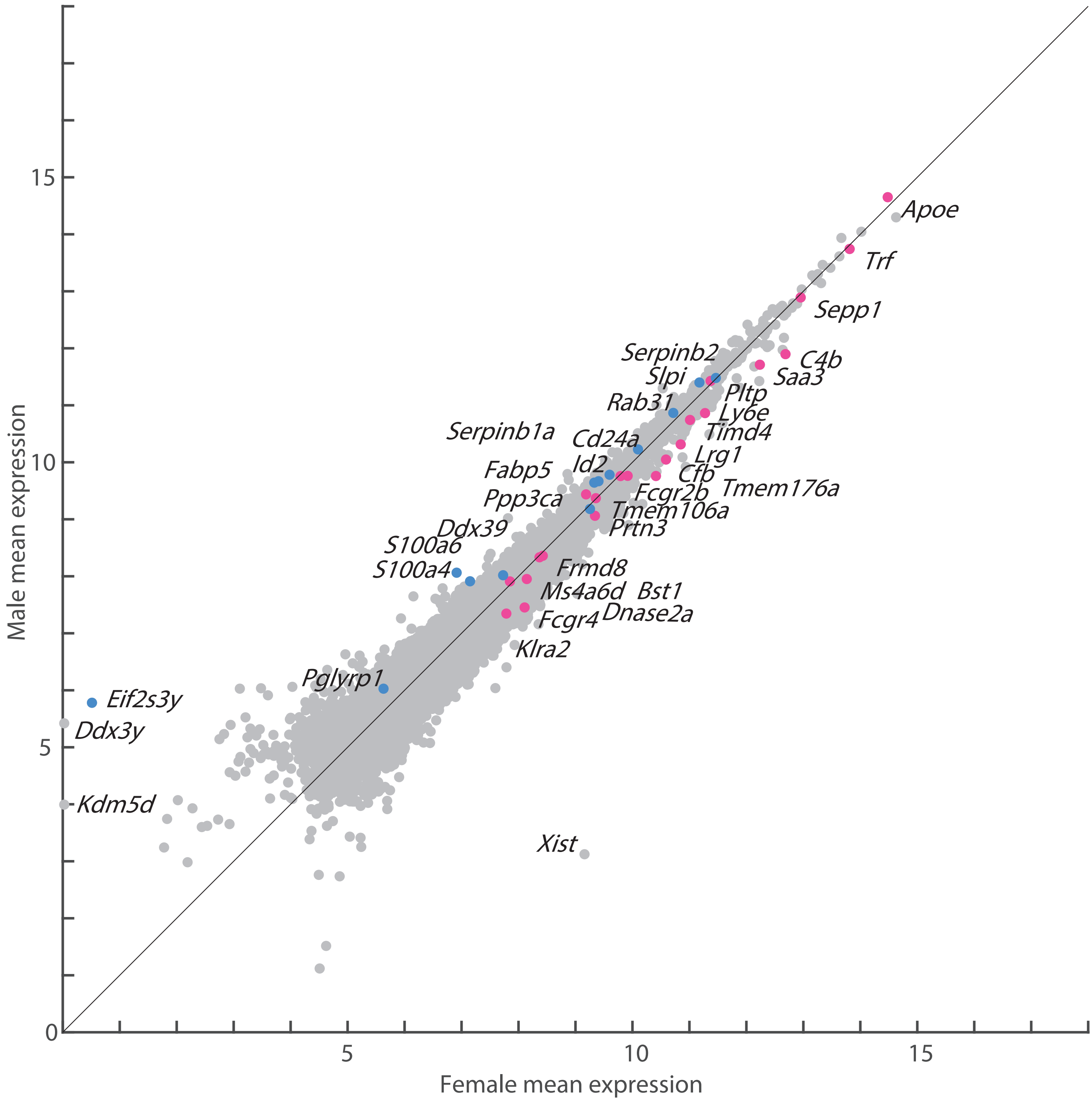

Supplementary Figure 5: MF-SDEGs are consistently different in the ATAC-seq matched RNA-seq data. Macrophage-specific SDEGs on male and female macrophages from dataset D. Mean expression in female (X axes) vs. male (Y axes), of all genes (gray), female SDEGs (pink) and male SDEGs (blue). SDEGs gene symbols are shown. Source data are provided as a Source Data file.

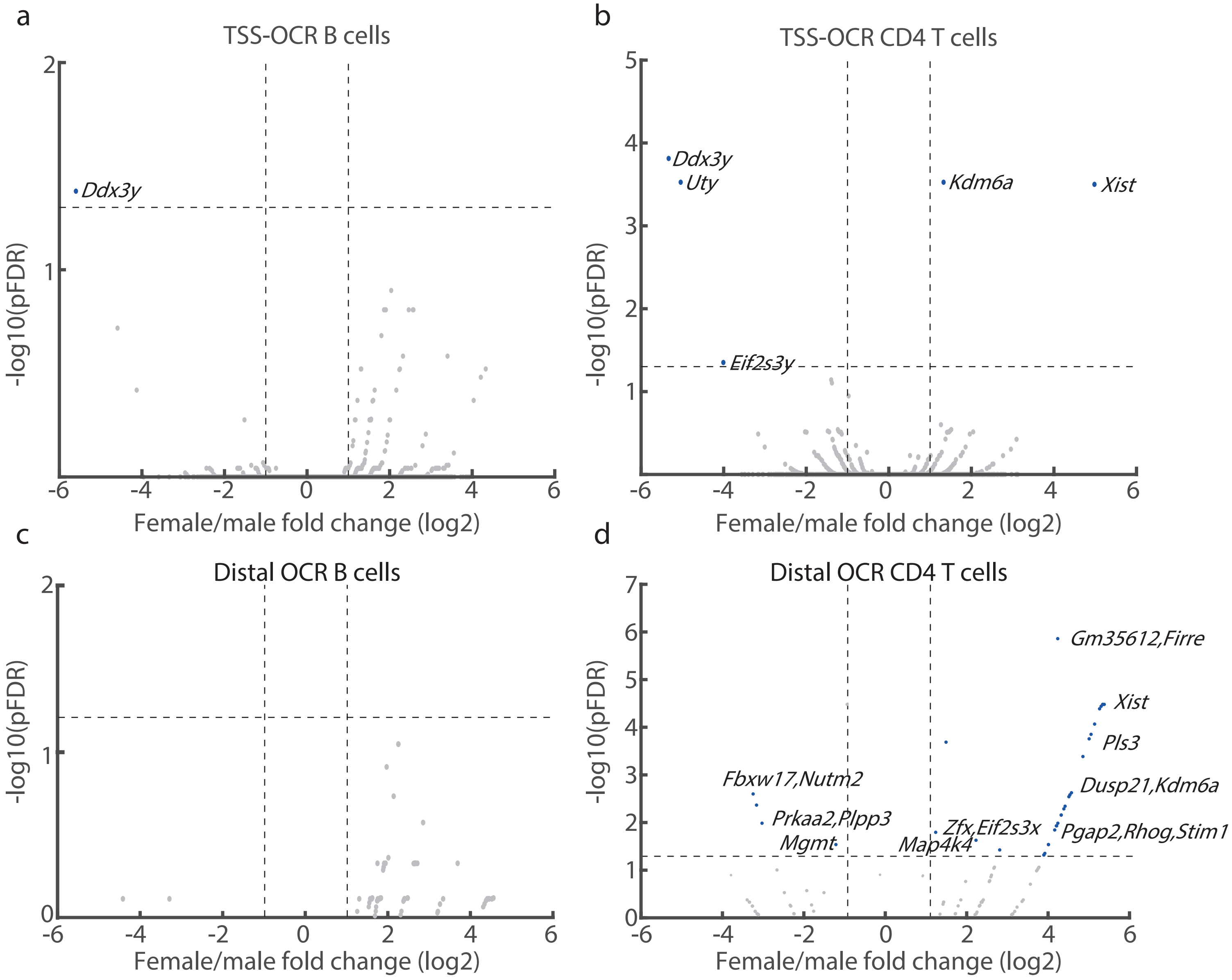

Supplementary Figure 6: Sexual differential accessibility is identified in immune cells. Volcano plots of female-male fold change versus pFDR (-log10) for differential OCRs (DOs) are shown in TSS regions for (a) B cells (1 DO) and (b) T4 cells (5 DOs). Distal enhancer regions are shown for (c) B cells (no DOs) and (d) T4 cells (34 DOs). Lines are marking the thresholds set for DOs pFDR < 0.05 and female-male fold change > 2). Source data for (a-d) are provided as a Source Data file.

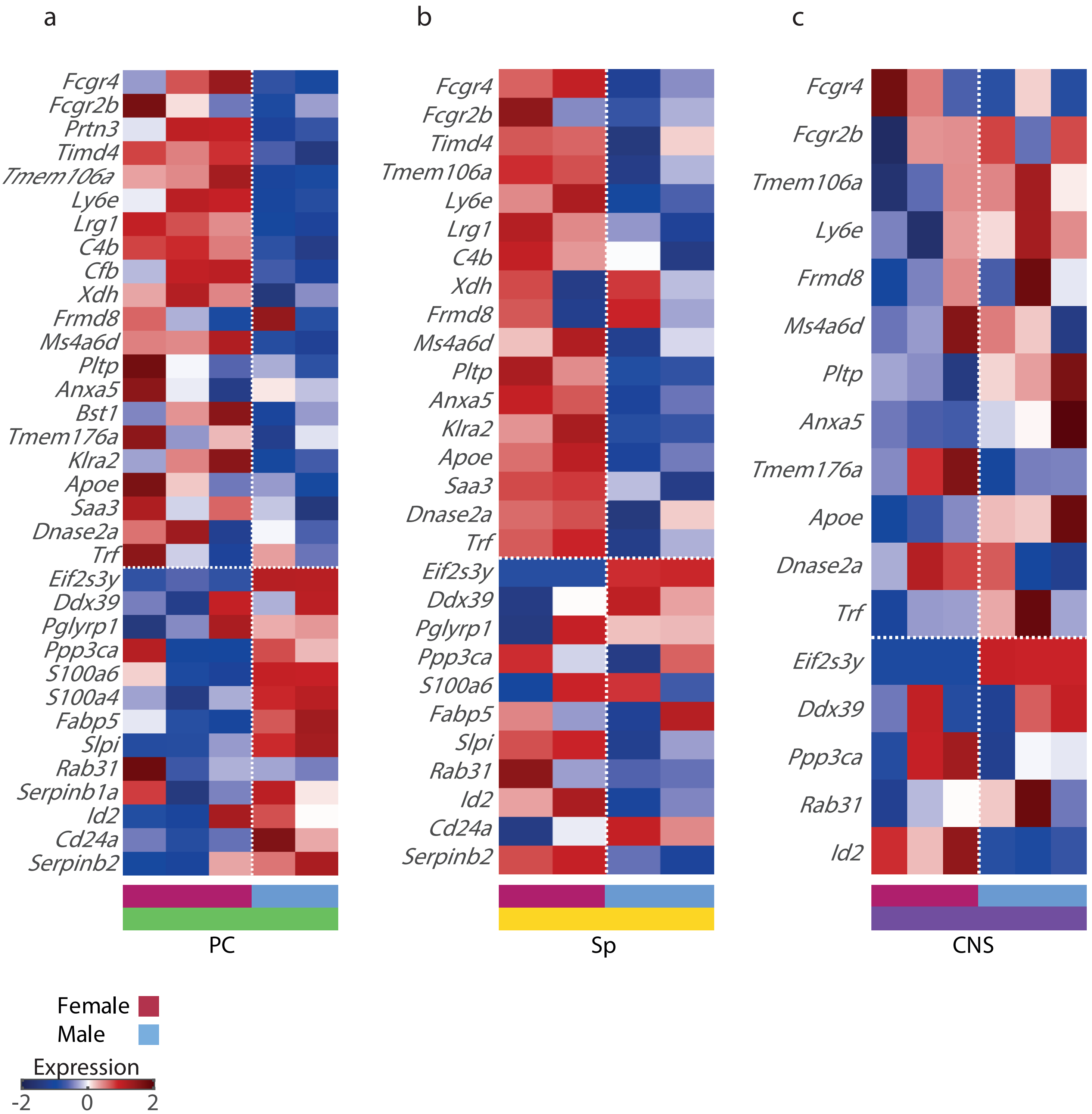

Supplementary Figure 7: MF-SDEGs identified in peritoneal cavity MFs display limited dimorphism in two other tissues. Heatmaps of relative expression levels of MF-specific male and female SDEGs identified in peritoneal cavity macrophages of datasets A and B showed in MF samples from: (a) peritoneal cavity (PC-MF, green), (b) spleen (Sp-MF, yellow) and (c) central nervous system (CNS, purple) (dataset C) are shown. Genes were sorted by mean male-female fold change. Only genes which passed expression filter per tissue are shown. Dotted horizontal white lines separate female from male up-regulated genes. Dotted vertical white lines separate female and male samples. Source data for (a-c) are provided as a Source Data file.

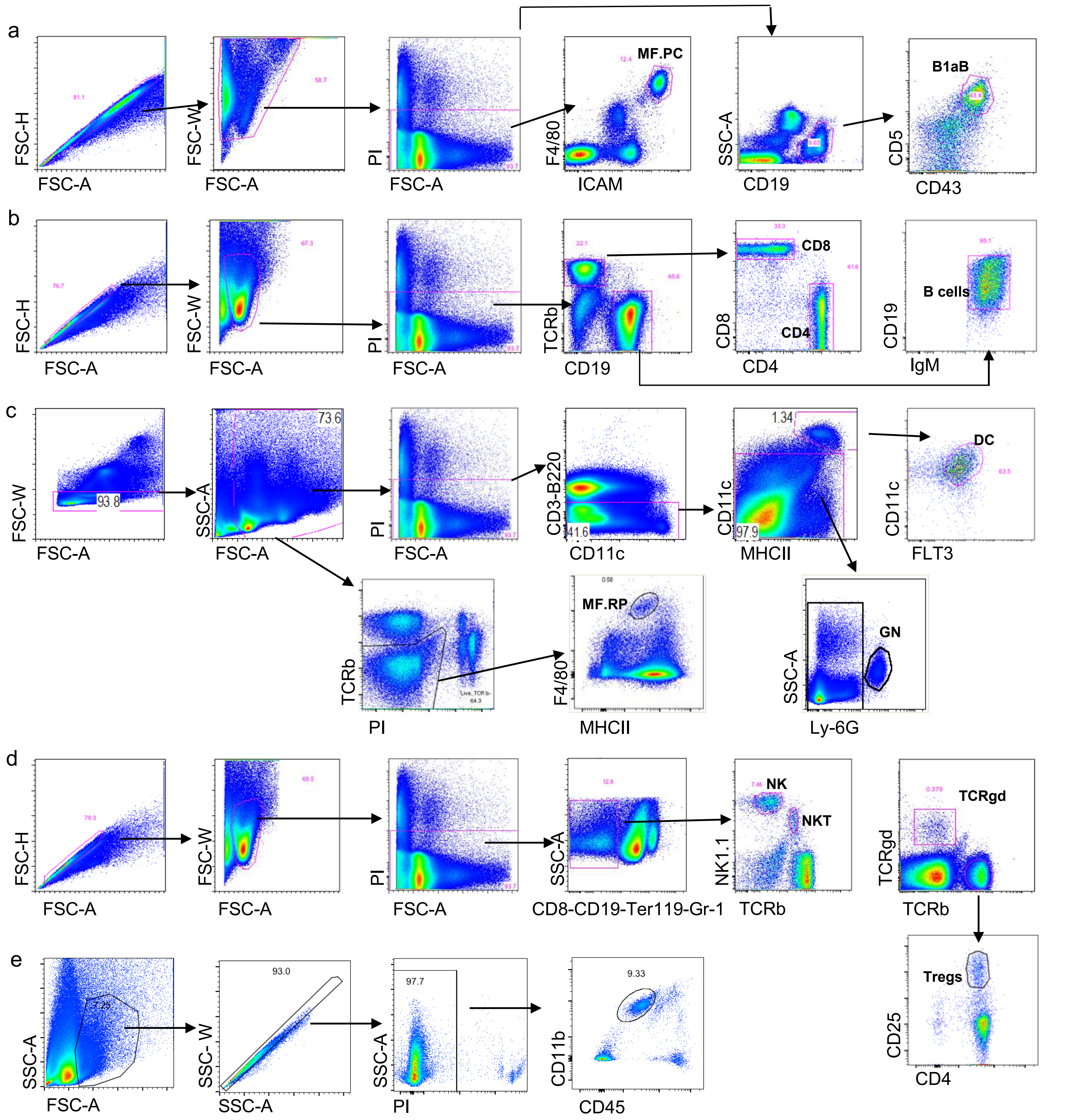

Supplementary Figure 8 : Gating strategies for cells sorted from C 57 BL/ 6 J. Gating strategy to sort: (a) Peritoneal B1aB (CD19+CD43+CD5+) cells and macrophages (F480+ICAM2+). (b) Splenic whole CD4 (CD19-TCRB+CD4+CD8-), whole CD8 (CD19-TCRB+CD4-CD8+) T cells, and B cells (CD19+IgM+TCRB-). (c) Splenic DC (TCRB-B220-CD11chi+ MHCII+Flt3+), Red Pulp macrophages (TCRB-MHCIIintF480hi), and Neutrophils (TCRB-B220-Ly6G+). (d) Splenic NK (CD8-CD19-Ter119-Gr1-NK1.1+TCRB-), NKT (CD8-CD19-Ter119-Gr1-NK1.1+TCRB+), gdT (CD8-CD19-Ter119-Gr1-TCRB-TCRgd+), and Tregs (CD8-CD19-Ter119-Gr1-TCRB+CD4+CD25+). (e) CNS macrophages (lin-PI-CD45intCD11 b+).

Supplementary Table 1 - Genes contributing to the sex effect according to the 12th component in PCA analysis		
**Gene Symbol**	**Locus**	**PCA Coeffitients (12th component)***
Ly6e	chr15:74954978-74966153	-0.080
Zbp1	chr2:173205320-173218922	-0.079
Pyhin1	chr1:173616117-173706395	-0.074
Tsix,Xist	chrX:103408377-103530703	-0.070
Ifi203,Mndal	chr1:173747219-173942492	-0.069
Bst2	chr8:71469193-71725681	-0.068
Cd48	chr1:171682042-171705257	-0.068
Tcrg	chr13:19162037-19341613	-0.067
Ahnak,Mir6367	chr19:8871495-9076926	-0.067
Tcrg-V1,Tcrg-V3	chr13:19162037-19341613	-0.066
Tcrg,Tcrg-C3	chr13:19162037-19341613	-0.064
Ctsc	chr7:88278092-88310875	-0.063
Tcrd,Trdd2	chr14:52744248-54273460	-0.062
Gm12184,Irgm1	chr11:48800229-48871683	-0.062
Plac8	chr5:100518308-100577093	-0.061
Ikbkb	chr8:22577074-22706583	-0.061
5830411n06rik	chr7:140247170-140300958	-0.059
Lpxn	chr19:12798534-12836203	-0.057
Sdf2	chr11:77982785-78255496	-0.057
Itgb7	chr15:102165181-102231995	-0.056
Ak035387	chr6:3240529-3346094	-0.056
M37286,V Delta 6, Tcr	chr14:52744248-54273460	-0.055
C920025e04rik,H2-T23	chr17:35743322-36121444	-0.055
Tcrg-C	chr13:19341778-19352759	-0.055
Gm8369,Ms4a4b,Ms4a4c,Ms4a4d,Ms4a6b,Ms4a6c	chr19:11343950-11574227	-0.055
Bscl2	chr19:8692294-8867843	-0.054

Psmb10	chr8:105900473-106054019	-0.054
Oas3	chr5:120511179-120824207	-0.053
Ak079938	chr19:11343950-11574227	-0.053
Olfr1396	chr11:49015873-49144967	-0.052
Rac1	chr5:143403819-143612674	-0.052
Crip1	chr12:113148777-113204076	-0.052
P2rx7	chr5:122619136-122692683	-0.051
S100a10	chr3:93555112-93628234	-0.051
Gm11625,Ifi35	chr11:101448298-101476521	-0.050
Igbp1	chrX:100494290-100518193	-0.050
Poldip3	chr15:83089505-83149336	0.050
Gm7964	chr7:83749753-83798317	0.051
Bcas2,Csde1,Nras	chr3:103020421-104220406	0.051
Sept2	chr1:93478992-93635752	0.052
Arhgef2,Gm25945	chr3:88607453-88775138	0.054
Edf1	chr2:25557842-25562082	0.055
Gm14494,Mapre1	chr2:153741286-153827070	0.059
Zfp263	chr16:3744098-3750788	0.060
Aim1l,Ubxn11	chr4:134052606-134245873	0.061
Dnase1,Trap1	chr16:4028912-4213496	0.064
Rbm25	chr12:83631235-83921934	0.064
Hnrnpdl	chr5:100033577-100039664	0.064
Gm13423,Yme1l1	chr2:23156170-23312403	0.065
Atrx	chrX:105797386-105929388	0.066
Abcg1	chr17:31057693-31277356	0.068
1700094d03rik,4933434e20rik,Ak217252	chr3:89977617-90068347	0.072
Nup210l	chr3:90072650-90473054	0.073
Eif2s3y	chrY:1010592-1286613	0.076
Nup210l,Tpm3	chr3:90072650-90473054	0.078
Ak033381,Tug1	chr11:3639784-3649042	0.100

* Positive coefficients for the male side, negative for the female

Supplementary Table 2 - Pan-immune sexually differentially expressed genes							
**Gene symbol**	**Locus**	**t-statistic**	**p value**	**pFDR**	**Fold change (log2)**	**Female mean (log2)**	**Male mean (log2)**
**Male>Female (pFDR < 0.2, FC(log2) > 1)**							
Eif2s3y	chrY:1010592-1286613	-73.08	1.27E-62	3.54E-59	-6.0	0.0	6.0
Ak007249	chr8:8613472-8787317	-5.82	2.18E-07	3.58E-05	-1.5	4.2	5.7
Inpp5f	chr7:128611327-128740620	-5.71	3.24E-07	5.01E-05	-0.9	4.4	5.3
Klf13	chr7:63879464-63938915	-6.59	1.02E-08	5.65E-06	-0.8	5.9	6.7
Ak031561, Set	chr2:30061772-30217564	-6.60	9.73E-09	5.65E-06	-0.7	4.3	5.1
Abhd8	chr8:71396847-71463657	-4.09	1.24E-04	5.33E-03	-0.7	2.9	3.5
Srsf2	chr11:116803399-116853124	-5.34	1.38E-06	1.32E-04	-0.7	4.6	5.2
Abcg1	chr17:31057693-31277356	-5.38	1.14E-06	1.20E-04	-0.6	5.4	6.1
Gm23547, Phb2	chr6:124663103-124718831	-3.97	1.87E-04	7.03E-03	-0.6	4.5	5.1
Kcnn4	chr7:24355262-24385364	-4.20	8.60E-05	4.10E-03	-0.6	3.0	3.6
Sephs2	chr7:127233254-127645111	-3.01	3.79E-03	6.62E-02	-0.6	3.1	3.7
**Female>Male (pFDR < 0.2, FC(log2) > 1)**							
Tsix,Xist	chrX:103408377-103530703	57.91	2.37E-56	3.31E-53	5.7	5.8	0.1
Oas3	chr5:120511179-120824207	6.01	1.03E-07	2.20E-05	0.6	3.3	2.7
Rsad2	chr12:26442742-26456461	4.50	2.94E-05	1.77E-03	0.6	2.5	1.9

Supplementary Table 3 - Functional enrichment of the top pan-immune sexually differentially expressed genes						
**Pathway name**	**Genes**	**Enrichment Score (ES)**	**Normalized ES**	**Nominal p-val**	**FDR q-val**	**Contributing genes**
**IFNα response**	34	0.77	2.57	<0.001	<0.001	*Ly6e, Irf7, Lgals3bp, Rsad2, Gbp2, Irf9, Rtp4, Tap1, Trafd1, Psmb9, Samd9l, Ifi27, Nmi, Psme1, Casp8, Ifi, M3, Elf1, Isg20, Lpar6, Casp1, Irf1, Sell, Bst2*
**IFNγ response**	84	0.62	2.48	<0.001	<0.001	*Oas3, Ly6e, Irf7, Lgals3bp, Stat1, Rsad2, Irf9, Zbp1, Rtp4, Oas2, Tap1, Trafd1, Samhd1, Psmb9, Samd9l, Nfkb1, Lcp2, Ifi27, Nmi, Psme1, Fas, Casp8, Ifitm3, Isg20, Cfh, Stat4, Socs3, Casp1, Pml, Irf1, Bst2, Il10ra, Cd38, Sppl2a, Epsti1, Psmb10, Myd88, Casp4, Xcl1, Psme2, Ptpn2, Cd40, Nfkbia, Gpr18, Irf2*
**Complement system**	63	0.49	1.88	0.01	0.18	*Irf7, Ctsb, Hspa5, Psmb9, Lcp2, Lamp2, Ctsc, Lgmn, Cd36, Cfh, Lrp1, Ctss, Casp1, C3, Irf1, Ctso, Pik3r5, Lyn, Prcp, Casp4, Dusp6, Fn1, Lta4h, Irf2, S100a13, F10*
**Allograft rejection**	85	0.43	1.72	0.03	0.19	*Irf7, Stat1, Gbp2, Tap1, Il2rg, Lcp2, Tlr1, Fas, Zap70, Fcgr2b, Ctss, Stat4, Tap2, Igsf6, Itgal, Cd28, Npm1, Cd2, Abi1, Capg, Lyn, Psmb10, Il16, Cd8a, Cd40, Cd7, Cxcr3, Klrd1, Nme1, Ncf4, Il15, Cd3g, Hcls1, Icam1, Cd86, Ifngr2, Ly86, Tpd52, Ccr2*
**Inflammatory response**	59	0.45	1.71	0.02	0.15	*Ly6e, Irf7, Rtp4, Nfkb1, Lcp2, Nmi, Tlr1, P2rx7, Cybb, Cd48, Irf1, Sell, Bst2, Il10ra, Rgs1, Pik3r5, Abi1, Il7r, Lyn*
**IL6 JAK-STAT3**	33	0.51	1.70	0.02	0.13	*Stat1, Irf9, Il2rg, Fas, Il13ra1, Cd36, Socs3, Irf1, Cbl, Cd38, Pik3r5, Myd88, Ptpn2, Il1r2*
**Coagulation**	35	0.49	1.67	0.03	0.12	*Ctsb, Lamp2, Arf4, Lgmn, Rac1, Cfh, Lrp1, C3, Ctso, Pef1, Dusp6, Fn1, S100a1, Lta4h, Gng12, S100a13, F10*

Supplementary Table 4 - Macrophage (MF)-specific sexually differentially expressed genes

Gene Symbol	Locus	t-statistic	p value	pFDR	Fold change (log2)	Female mean (log2)	Male mean (log2)
**Male MF-SDEGs: MF Male>Female (pFDR < 0.2, FC(log2) < 1)**							
Eif2s3y	chrY:1010592-1286613	-22.101	3.52E-06	7.24E-03	-5.8	9.2	9.8
S100a4	chr3:90603769-90611780	-5.005	4.09E-03	0.131	-1.3	5.7	6.7
Cd24a,F830002l21rik	chr10:43578658-43669646	-4.104	9.31E-03	0.182	-1.0	4.8	5.4
S100a6	chr3:90612893-90614414	-7.992	4.95E-04	0.121	-1.0	6.9	7.8
Pglyrp1	chr7:18884629-18916897	-4.613	5.77E-03	0.152	-1.0	6.7	7.4
Mir7678,Slpi	chr2:164296517-164443255	-4.748	5.11E-03	0.144	-1.0	6.9	7.5
Id2	chr12:24974824-25096092	-4.925	4.38E-03	0.131	-0.9	5.2	5.8
Ppp3ca	chr3:136626390-136939574	-8.482	3.74E-04	0.110	-0.8	11.2	12.2
Fabp5	chr3:10012584-10016610	-4.123	9.14E-03	0.182	-0.8	5.4	6.1
Serpinb1a,Serpinb1c	chr13:32802029-32898140	-4.951	4.28E-03	0.131	-0.7	7.2	8.4
Ddx39	chr8:83715176-83829382	-4.596	5.86E-03	0.152	-0.7	5.9	6.9
Rab31,Vapa	chr17:65580052-65772752	-5.080	3.83E-03	0.131	-0.6	4.9	5.7
Gm24989	chr11:77216274-77460222	-6.094	1.72E-03	0.121	-0.6	4.1	5.1
Oaz1-Ps	chr17:17334933-17346394	-5.729	2.27E-03	0.121	-0.6	4.7	5.4
Serpinb2	chr1:107501060-107622313	-5.197	3.48E-03	0.131	-0.6	0.0	5.8
**Female MF-SDEGs: MF Female>Male (pFDR < 0.2, FC(log2) > 1)**							
Saa3	chr7:46711997-46715676	5.699	2.32E-03	0.121	1.8	13.5	11.7
Fcgr4	chr1:170960558-171046296	4.169	8.75E-03	0.182	1.5	6.8	5.3
Lrg1	chr17:56079624-56290541	5.529	2.65E-03	0.121	1.3	7.7	6.4
Lrg1,Sema6b	chr17:56079624-56290541	7.760	5.68E-04	0.121	1.3	9.0	7.7
Cfb,Mir6972	chr17:34850390-34862514	4.259	8.03E-03	0.176	1.2	7.9	6.7
Apoc2,Apoc4	chr7:19505791-19824279	5.339	3.09E-03	0.126	1.2	8.6	7.4
Apoe	chr7:19505791-19824279	6.068	1.76E-03	0.121	1.1	14.9	13.8
Ly6e	chr15:74954978-74966153	5.552	2.61E-03	0.121	1.1	8.6	7.5
Prtn3	chr10:79697304-80369637	4.045	9.87E-03	0.188	1.0	8.4	7.4
C4a,C4b	chr17:34589805-34836927	5.734	2.26E-03	0.121	1.0	9.0	8.0
Stxbp3a	chr3:108788559-108911710	5.164	3.57E-03	0.131	0.9	6.8	5.8

Ms4a6d	chr19:11586605-11604811	7.037	8.95E-04	0.121	0.9	6.4	5.5
Timd4	chr11:46810700-46844333	4.517	6.30E-03	0.155	0.9	8.1	7.2
Fcgr2b	chr1:170960558-171046296	4.114	9.23E-03	0.182	0.9	8.1	7.2
Sepp1	chr15:3268546-3280585	5.738	2.25E-03	0.121	0.9	10.9	10.1
Pltp	chr2:164769892-164919953	5.449	2.83E-03	0.124	0.8	9.4	8.6
Tmem106a	chr11:101552106-101599256	8.554	3.60E-04	0.110	0.7	6.5	5.7
Tmem176a	chr6:48841482-48847106	4.862	4.62E-03	0.135	0.7	7.9	7.2
Anxa5,Mir7009	chr3:36445485-36475887	4.245	8.13E-03	0.176	0.7	6.7	6.0
Klra2	chr6:131212997-131247362	4.918	4.40E-03	0.131	0.7	5.8	5.1
Ak172267	chr13:120034604-120052227	6.745	1.09E-03	0.121	0.7	5.5	4.9
Trf	chr9:103208872-103305082	4.981	4.17E-03	0.131	0.6	12.2	11.5
Dnase2a,Loc100503676	chr8:84908618-84937353	6.902	9.78E-04	0.121	0.6	5.5	4.9
Bst1	chr5:43818871-43912380	5.668	2.38E-03	0.121	0.6	5.6	5.0
Frmd8,Malat1	chr19:5773783-5912866	8.494	3.72E-04	0.110	0.6	8.8	8.2
Xdh	chr17:73804794-73951492	5.857	2.06E-03	0.121	0.6	6.9	6.3

Supplementary Table 5 - Differentially accessible regions in TSS DOs and distal enhancers DOs				
**Gene Symbol**	**pFDR**	**Fold Change (log2)**	**Female mean**	**Male mean**
**MF TSS DOs**				
Xist	7.11E-08	3.146	3.770	0.625
Kdm6a	5.33E-07	1.463	7.004	5.540
Cfp	1.86E-04	1.402	6.058	4.655
Eif2s3x	0.008	1.374	4.869	3.495
Bckdhb	0.037	-1.094	4.682	5.776
Ift74	0.037	-1.265	3.845	5.110
Ncam2	0.008	-2.148	1.001	3.149
Ddx3y	4.39E-15	-4.173	0.000	4.173
**T4 TSS DOs**				
Xist	3.16E-04	4.982	4.982	0.000
Kdm6a	2.98E-04	1.326	7.645	6.318
Eif2s3y	0.045	-4.008	0.000	4.008
Uty	2.98E-04	-5.041	0.000	5.041
Ddx3y	1.54E-04	-5.333	0.000	5.333
**B cell TSS DOs**				
Ddx3y	0.042	-5.599	0.000	5.599
**MF distal enhancer DOs**				
Gm35612,Firre	4.37E-29	5.668	5.668	0.000
Gm35612,Firre	1.32E-28	5.600	5.600	0.000
Gm35612,Firre	9.07E-28	5.511	5.511	0.000
Gm35612,Firre	1.80E-26	5.383	5.383	0.000
Gm35612,Firre	7.38E-25	5.225	5.583	0.358
Gm35612,Firre	6.60E-22	4.932	4.932	0.000
Gm35612,Firre	3.69E-21	4.851	4.851	0.000
4933407K13Rik,Pls3	2.04E-20	4.769	4.769	0.000
Gm35612,Firre	3.72E-20	4.737	4.737	0.000

Gm35612,Firre	1.19E-18	4.575	4.575	0.000
Gm35612,Firre	3.38E-17	4.412	4.412	0.000
Gm35612,Firre	1.71E-15	4.215	4.215	0.000
Gm35612,Firre	2.90E-15	4.185	4.185	0.000
4933407K13Rik,Pls3	4.46E-15	4.159	4.159	0.000
Gm35612,Firre	5.02E-15	4.149	4.149	0.000
Gm35612,Firre	1.22E-14	4.101	4.101	0.000
4933407K13Rik,Pls3	2.30E-14	4.065	4.065	0.000
Gm35612,Firre	5.51E-12	3.766	4.124	0.358
Gm35612,Firre	7.57E-12	3.745	4.503	0.758
Gm35612,Firre	9.85E-09	3.314	3.939	0.625
Gm35612,Firre	6.84E-07	3.027	4.120	1.093
NA	4.02E-06	2.899	3.657	0.758
Dusp21,Kdm6a	1.31E-07	2.399	4.201	1.802
Gm35612,Firre	4.43E-06	2.205	4.119	1.914
NA	2.29E-03	1.483	6.306	4.823
Atp11c,Mir505	3.37E-02	1.461	5.254	3.793
Araf,Syn1,Mir5617,Timp1	3.37E-02	1.459	5.454	3.995
Mgmt	3.37E-02	-1.464	3.670	5.133
NA	6.21E-03	-2.303	0.971	3.274
**T4 distal enhancer DOs**				
Gm35612,Firre	3.35E-05	5.384	5.384	0.000
Gm35612,Firre	3.35E-05	5.349	5.349	0.000
Tsx,Tsix,Xist,Jpx,Gm9159	3.35E-05	5.345	5.345	0.000
Gm35612,Firre	3.67E-05	5.308	5.308	0.000
Gm35612,Firre	4.14E-05	5.270	5.270	0.000
Gm35612,Firre	8.67E-05	5.148	5.148	0.000
4933407K13Rik,Pls3	1.42E-04	5.060	5.060	0.000
4933407K13Rik,Pls3	1.77E-04	5.014	5.014	0.000
Gm35612,Firre	4.16E-04	4.861	4.861	0.000
4933407K13Rik,Pls3	2.40E-03	4.579	4.579	0.000

Gm35612,Firre	2.65E-03	4.541	4.541	0.000
Gm35612,Firre	2.90E-03	4.516	4.516	0.000
Dusp21,Kdm6a	4.59E-03	4.422	4.422	0.000
Gm35612,Firre	5.22E-03	4.393	4.393	0.000
Gm35612,Firre	7.03E-03	4.328	4.328	0.000
Gm35612,Firre	7.03E-03	4.327	4.327	0.000
Gm35612,Firre	1.04E-02	4.245	4.245	0.000
Gm35612,Firre	1.40E-06	4.242	5.467	1.225
Tsx,Tsix,Xist,Jpx,Gm9159	1.19E-02	4.214	4.214	0.000
Gm35612,Firre	1.19E-02	4.209	4.209	0.000
Gm35612,Firre	1.45E-02	4.168	4.275	0.107
Tsx,Tsix,Xist,Jpx,Gm9159	2.93E-02	4.015	4.015	0.000
Pgap2,Rhog,Stim1	2.93E-02	4.013	4.013	0.000
5530601H04Rik,Pbdc1,M	4.48E-02	3.925	4.247	0.322
Dusp21,Kdm6a	4.84E-02	3.902	4.010	0.107
Gm35612,Firre	4.84E-02	3.900	4.731	0.831
Gm35612,Firre	3.79E-02	2.816	4.661	1.844
Gm3646,1700066B17Rik,	2.38E-02	2.234	5.732	3.498
NA	2.08E-04	1.498	7.297	5.799
Zfx,Eif2s3x,Klhl15,Mir76	1.62E-02	1.244	6.926	5.682
Mgmt	2.93E-02	-1.208	5.416	6.624
Prkaa2,Plpp3	1.04E-02	-3.023	1.741	4.763
NA	4.35E-03	-3.157	1.442	4.599
Fbxw17,Nutm2	2.54E-03	-3.241	1.984	5.225

Supplementary Table 6 - Functional enrichment of the genes passing ANOVA sex*tissue interaction effect - tissue macrophages

Sex	Tissue	Enriched pathway	p value	Involved genes	Annotation description
Female	Spleen	Apoptosis	1.55E-04	*App, Atf3, Bax, Ccnd1, H1f0, Hmox1, Jun, Ppt1*	Genes mediating programmed cell death (apoptosis) by activation of caspases.
		Tnfa signaling via nfkb	5.11E-04	*Atf3, Ccnd1, Hes1, Jun, Ninj1, Ripk2, Serpinb2, Zfp36*	Genes regulated by NF-kB in response to TNF.
		Complement	1.08E-03	*C1qc, Ctss, Fn1, Irf2, Lgmn, Ppp4c, Serpinb2*	Genes encoding components of the complement system, which is part of the innate immune system.
		Androgen response	1.93E-03	*Ccnd1, H1f0, Insig1, Myl12a, Vapa*	Genes defining response to androgens.
		P53 pathway	2.57E-03	*App, Atf3, Bax, Hmox1, Ifi30, Jun, Ninj1*	Genes involved in p53 pathways and networks.
Male	Spleen	E2f targets	2.73E-07	*Anp32e, Dnmt1, Espl1, Lbr, Mlh1, Orc2, Plk4, Rad21, Rfc1, Ssrp1, Tfrc, Ubr7*	Genes encoding cell cycle related targets of E2F transcription factors.
		Heme metabolism	3.85E-06	*Endod1, Fech, Narf, Ranbp10, Rnf123, Selenbp1, Slc4a1, Spta1, Tfrc, Trak2*	Genes involved in metabolism of heme (a cofactor consisting of iron and porphyrin) and erythroblast differentiation.
	CNS	Epithelial mesenchymal transition	4.42E-04	*Col4a1, Fn1, Igfbp3, Itgb1*	Genes defining epithelial-mesenchymal transition, as in wound healing, fibrosis and metastasis.

Supplementary Note 1 – Power analysis for the pan-immune and cell type specific comparisons

We performed the following power analysis for fdr, taking into account the multiple testing issue we face here, using the locfdr R package[4]. We run the locfdr R command with nulltype=0 (using the theoretical null for z-score transformed values), and pct=0.001 (excluding 0.1% tails for estimating the non-null distribution). The function reports *Efdr*, an estimate for the expected local false discovery rate at which a non-null gene is detected over all non-null genes (i.e. genes hypothesized to have a true sex-related effect). For the pan-Immune analysis, we got $\widehat{Efdr} = 0.38$, indicating moderate power, and for the cell type specific analysis on macrophages $\widehat{Efdr} = 0.45$, indicating lower power to detect cell-type specific effects in macrophages.

While *Efdr* measures the average fdr level for detection, we also used the locfdr package to plot a curve of fdr vs. power as a function of p-value threshold, shown in Note Figure 1 (right) for the pan-Immune analysis and in Note Figure 2 (right) for the cell-type specific analysis. For example, at fdr level 0.2, we have power to detect ~0.31 of the non-null genes in the pan-Immune analysis and ~0.11 of the macrophage-specific genes.

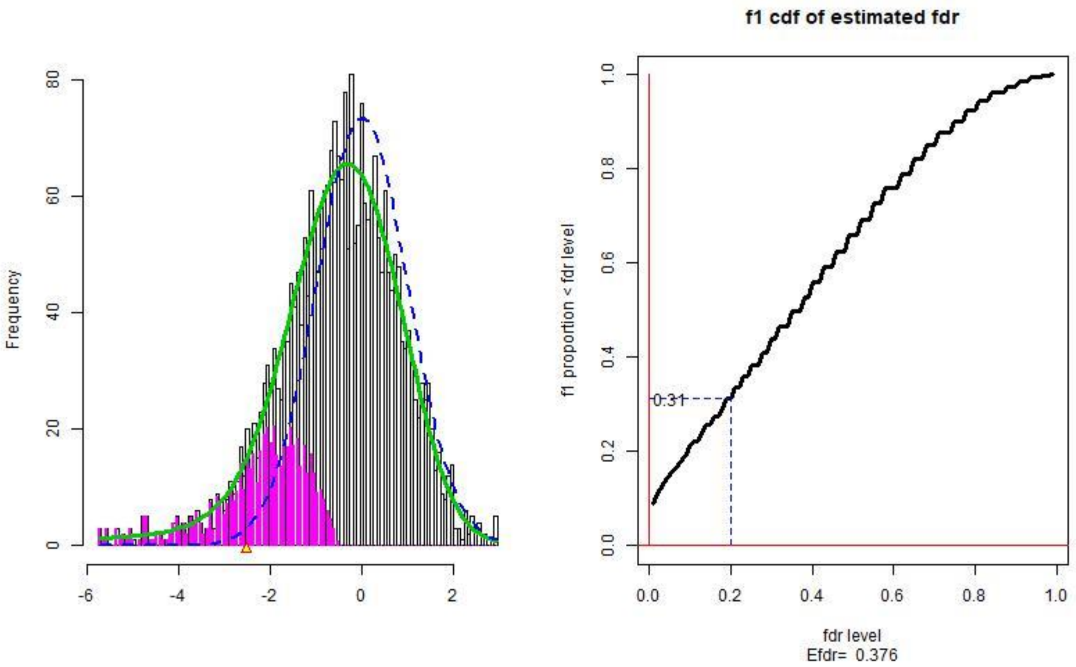

Note Figure 1: Power diagnostic for the pan immune cell analysis. Left: A local fdr analysis of the 2786 genes shows a significant left tail in the z-score distribution of genes (pink histogram bars) compared to the null distribution (black and white bars and dashed blue curve), indicating significant genes with higher expression in females vs. males. The yellow triangle indicates the fdr=0.2 cutoff, such that the proportion of the nulls at this cutoff is ~0.2. The overall proportion of non-null genes, i.e. genes differentially expressed between males and females 1-p_0 is estimated using two methods at between 0.6% (Central Matching Estimate) and 1.3% (Maximum Likelihood Estimate). Right: Proportion of nun-null genes detected (y-axis) vs. desired fdr level (x-axis). For example, at *fdr*=0.2 level we expect to detect ~31% of non-null genes.

We focused the cell-type specific power analysis on macrophage, which showed the strongest effect among the 11 cell types. Other cell types show none or negligible effects, and therefore larger sample sizes are needed in order to detect possible additional effects and get a reliable power analysis.

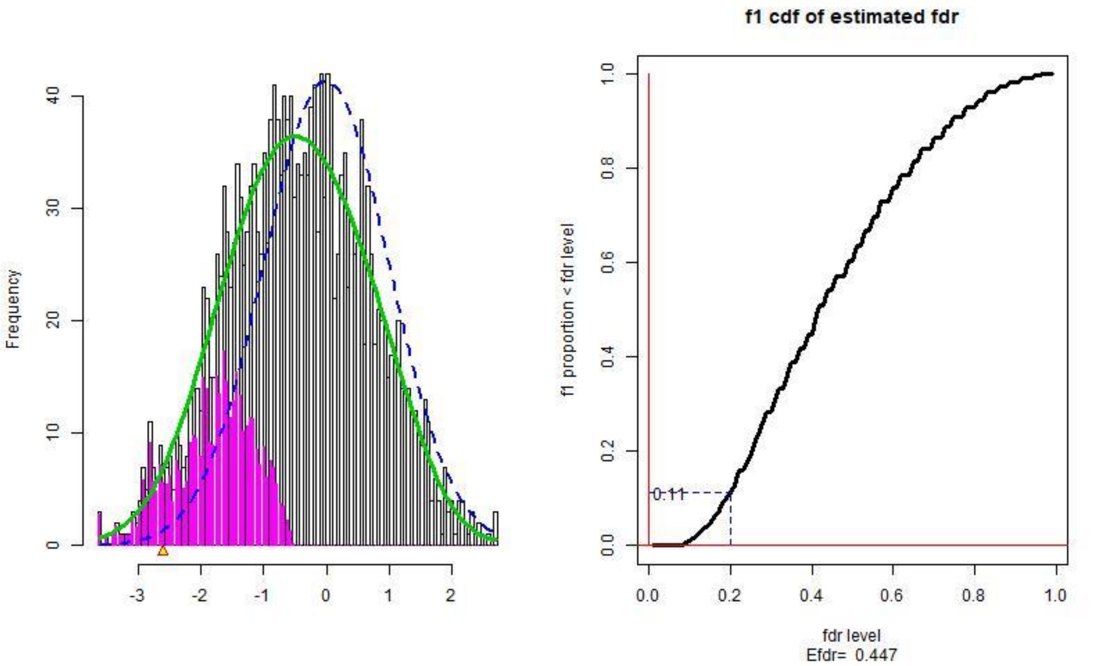

Note Figure 2: Power diagnostic for the macrophage-specific analysis. Left: A local fdr analysis of the 2055 genes shows a significant left tail in the z-score distribution of genes (pink histogram bars) compared to the null distribution (black and white bars and dashed blue curve), indicating significant genes with higher expression in females vs. males. Right: Proportion of nun-null genes detected (y-axis) vs. desired fdr level (x-axis). For example, at *fdr*=0.2 level we expect to detect ~11% of non-null genes.

Finally, we analyzed how increasing the sample size is expected to increase our power. Note Table 1 shows the reduction in expected fdr as we increase the sample size by 1.5-fold to 10-fold, showing improvement in power for both analyses. Similarly, if we fix the fdr level at 0.2 as in the current study, the table show the improvement in power for the pan immune and macrophage analysis.

**Relative sample size**	**1**	**1.5**	**2**	**3**	**4**	**5**	**10**
Pan-Immune $\widehat{Efdr}$	0.38	0.30	0.24	0.18	0.14	0.12	0.06
Cell-Type Specific $\widehat{Efdr}$	0.45	0.36	0.30	0.24	0.20	0.18	0.11
Pan-Immune $Power(fdr = 0.2)$	0.31	0.48	0.59	0.70	0.76	0.81	0.90
Cell-Type Specific $Power(fdr = 0.2)$	0.11	0.36	0.47	0.60	0.67	0.71	0.83

Note Table 1: Estimated decrease in expected fdr ($\widehat{Efdr}$), and power at *fdr=0.2* level as a function of relative sample size for the pan Immune and cell-type specific analysis (1 refer to sample size used in the current study).

Supplementary Note 2 – Disease context and mouse phenotypes for the 41 macrophage specific sexually differentially expressed genes (SDEGs)

Genes are referred by their murine gene symbols. Human orthologues were identified by Ensembl Compara orthology (https://www.ensembl.org/index.html).

Data curated from:

1. *Pubmed* (https://www.ncbi.nlm.nih.gov/pubmed)
2. *OMIM* (https://www.omim.org/)
3. *GeneCards* (https://www.genecards.org/)
4. *The Jackson Laboratory* (https://www.jax.org/)

Male SDEGS

Eif2s3y

Mouse phenotype: Spermatogenesis and suppression of pluripotency maintenance of embryonic stem cells[5].

S100a4

Human disease: Tumor progression and metastasis in various cancers[6-9].

Cd24a, F830002l21rik

Human disease: CD24 human polymorphism associated with multiple sclerosis[10], tumor invasiveness and metastatic potential in hepatocellular carcinoma[11].

S100a6

Human disease: Tumor progression and metastasis in various cancers[12, 13].

Pglyrp1

Human disease: Antitumor activity in cell lines[14]; Stimulates lymphocyte differentiation toward antitumor activity[15].

Mouse phenotype: Pglyrp1-KO mice have increased susceptibility to induced colitis[16].

Mir7678, Slpi

Human disease: Oral mucosal response to HIV-1[17].

Mouse phenotype: *Slpi*-KO mice show impaired wound healing[18].

Id2

Human disease: endometriosis[19]; Neuroblatoma in children[20]

Mouse phenotype: *Id2*-KO have multiple alterations in the circadian system[21], *Id2*/*Id3* double-knockout mice show rapid lymphoma development[22]

Ppp3ca

Human disease: Mutation associated with neurodevelopmental disease and seizures[23]

Mouse phenotype: transgenic mice with activated PPP3ca in the heart developed cardiac hypertrophy[24]; *Ppp3ca* -KO generates defective antigen specific T cell response in-vivo[25].

Fabp5

Human disease: Cervical cancer growth and metastasis[26].

Mouse phenotype: ***Fabp5*** deficiency result in decreased keratin 1 expression through downregulation of NF-κB activity and potentially related to psoriasis[27].

Serpinb1a, Serpinb1c

Human disease: Type 2 diabetes[28] and β-cell increased proliferation in humans, mice, and zebrafish[29].

Ddx39

Human disease: Hepatocellular carcinoma[30]; Neuroblastoma (inferred from human cell line)[31].

Rab31, Vapa

Human disease: Cancer progression, proliferation, and apoptosis (inferred from human cell lines)[32, 33]**.**

Mouse phenotype: *Rab31* knockdown suppresses tumor growth[32].

Gm24989

N/A

***Oaz1*-Ps**

N/A

Serpinb2

Human disease: Asthma[34]**;** Human Polymorphism also associated with lupus[35]; Decreased virus replication, including influenza[36] and hepatitis C[37].

Mouse phenotype: Inflammatory conditions[38] and suppression of Th1-promoting cytokine production[39].

<u>Female SDEGs</u>

Saa3

Mouse phenotype: deficient mice developed obesity, abnormal lung development and homeostasis and impaired cytokine production in CD4 and CD8 T cells[40].

Fcgr4

Mouse phenotype: Contributes to arthritis in mouse[41]. *FcγRIV*-deficient mice are impaired in IgG2a- and IgG2b-dependent effector functions[42, 43].

Lrg1

Human disease: Granulocyte differentiation (inferred from cell lines) [44].

Mouse phenotype: reduction in pathological ocular angiogenesis[45].

Lrg1, Sema6b

Human disease: Tumor differentiation and metastasis in gastric cancer[46]; breast cancer progression[47]

Cfb, Mir6972

Human disease: Complement Factor B Deficiency[48]; Age-Related Macular Degeneration[49].

Apoc2, Apoc4

Human disease: Hypertriglyceridaemia[50]; Apolipoprotein C-II Deficiency[51].

Mouse phenotype: transgenic mice expressing the human apoc2 gene develop hypertriglyceridemia and have delayed clearance of VLDL-triglyceride[52].

Apoe

Human disease: Hyperlipoproteinemia Type III[53]; Cardiovascular Disease[54, 55]; Alzheimer Disease 2[55-57]

Mouse phenotype: Apoe deficient mice show increased plasma cholesterol[58].

Ly6e

Human disease: Viral infection diseases (inferred from cell lines) [59, 60]

Prtn3

Human disease: Autoantigen mediated immune disregulation[61, 62]

Mouse phenotype: knockdown cause delayed neutrophil death[63]

C4a,C4b

Human disease: Deficiency associated with the risk to develop systemic autoimmune diseases[64]

Mouse phenotype: C4b deficient mice have increased susceptibility to lethal infection by Group B streptococci[65] and profound defect in antibody response to T cell dependent antigens[66]

Stxbp3a

Human disease: Down-regulated in obesity[67]; Insulin release regulation[68]

Mouse phenotype: Increased susceptibility for severe glucose intolerance in heterozygous knockout mice[69].

Ms4a6d

Human disease: Polymorphism of the human ortholog (*MS4A6A*) influencing Alzheimer's disease specific brain structures[70].

Timd4

Human disease: Renal cell carcinoma[71].

Mouse phenotype: *Timd4* expression level modulating immune pathways and inhibits nitric oxide secretion in murine macrophages[72].

Fcgr2b

Human disease: Polymorphism associated with systemic lupus erythematosus[73]. Expression levels are associated with Lymphocytic leukemia[74].

Mouse phenotype: Knockout mice develop spontaneous Lupos[75, 76].

Sepp1

Human disease: Polymorphism associated with breast cancer[77, 78]

Mouse phenotype: Neurological dysfunctions[79] and neurodegeneration[80] in *Sepp1* deficient mice.

Pltp

Human disease: PLTP activity is positively correlated with coronary artery disease[81], and the metabolic syndrome[82].

Mouse phenotype: *Pltp* deficiency in mice cause alterations in cholesterol metabolism[83].

Tmem106a

Human disease: Expression level associated with tumor suppression of lung[84], and kidney cancer[85].

Mouse phenotype: The protein is involved in peritoneal macrophages activation[86].

Tmem176a

Mouse phenotype: Expression associated with tumor suppression in colorectal[87], and hepatocellular[88] carcinoma in mouse models.

Anxa5, Mir7009

Human disease: Expression associated with colon cancer[89]. The M2 haplotype of the gene is associated with pregnancy loss[90, 91].

Klra2, Ly49b

Mouse phenotype: This type II lectin receptor binds class I MHC without peptide-specificity, function as a Natural-Killer receptor in mice[92].

Ak172267

Trf

Human disease: Correlated with tremor phenotype of Parkinson's disease[93]. Polymorphism associated with decreased risk for Ischemic Stroke[94].

Dnase2a, Loc100503676

Human disease: Deficiency associated with auto-inflammation[95]. Nonsynonymous SNPs causing los-of-function are associated with autoimmunity[96].

Mouse phenotype: *DNaseII* deficient mice develop chronic polyarthritis, which resembles rheumatoid arthritis[97].

Bst1

Human disease: Polymorphism associated with susceptibility to Parkinson's disease[98].

Mouse phenotype: anxiety-related and depression-like behaviors in deficient mice[99].

Frmd8, Malat1

Human disease: *Frmd8* regulates inflammatory and growth factor signaling in human and mouse macrophages[100]. *Malat1* is associated with non-small cell lung cancer and diabetes[101].

Mouse phenotype: *Frmd8*-KO are viable and fertile; *Malat1*-KO exhibit increased susceptibility to ischemic brain injury with increased cerebral infarction size, worse neurological scores and sensorimotor dysfunction, and increased expression of pro-apoptotic and pro-inflammatory factors in brain than wild-type controls.

Xdh

Human disease: Expression negatively associated with bad prognosis in neoplasms of the breast, liver, gastrointestinal tract, and kidney[102]. Polymorphism associated with gastric cancer survival[103].

Mouse phenotype: Nonsense mutation in *Xdh* gene cause early-onset renal failure in mice[104].

Supplementary references

1. Mostafavi, S. *et al*. Parsing the Interferon Transcriptional Network and Its Disease Associations. *Cell* **164**, 564-578 (2016).

2. Chiche, L. *et al*. Modular transcriptional repertoire analyses of adults with systemic lupus erythematosus reveal distinct type I and type II interferon signatures. *Arthritis Rheumatol.* **66**, 1583-1595 (2014).

3. Lee, H. M., Sugino, H., Aoki, C. & Nishimoto, N. Underexpression of mitochondrial-DNA encoded ATP synthesis-related genes and DNA repair genes in systemic lupus erythematosus. *Arthritis Res. Ther.* **13**, R63 (2011).

4. Efron, B. Size, power and false discovery rates. *The Annals of Statistics* **35**, 1351-1377 (2007).

5. Li, N. *et al*. EIF2S3Y suppresses the pluripotency state and promotes the proliferation of mouse embryonic stem cells. *Oncotarget* **7**, 11321-11331 (2016).

6. Egeland, E. V. *et al*. Prognostic significance of S100A4-expression and subcellular localization in early-stage breast cancer. *Breast Cancer Res. Treat.* **162**, 127-137 (2017).

7. Stewart, R. L. *et al*. S100A4 drives non-small cell lung cancer invasion, associates with poor prognosis, and is effectively targeted by the FDA-approved anti-helminthic agent niclosamide. *Oncotarget* **7**, 34630-34642 (2016).

8. Zhou, Y. *et al*. Overexpression of S100A4 protein may be associated with the development and progression of pancreatic cancer. *J Cancer Res Ther* **14**, S15-S166 (2018).

9. Zhang, J. *et al*. S100A4 promotes colon inflammation and colitis-associated colon tumorigenesis. *OncoImmunology* **7**, e1461301 (2018).

10. Zhou, Q. *et al*. CD24 is a genetic modifier for risk and progression of multiple sclerosis. *Proc. Natl. Acad. Sci. U. S. A.* **100**, 15041-15046 (2003).

11. Yang, X. *et al*. CD24 is a novel predictor for poor prognosis of hepatocellular carcinoma after surgery. *Clin. Cancer Res.* **15**, 5518-5527 (2009).

12. Maelandsmo, G. M. *et al*. Differential expression patterns of S100A2, S100A4 and S100A6 during progression of human malignant melanoma. *Int. J. Cancer* **74**, 464-469 (1997).

13. Ilg, E. C., Schäfer, B. W. & Heizmann, C. W. Expression pattern of S100 calcium-binding proteins in human tumors. *Int. J. Cancer* **68**, 325-332 (1996).

14. T N Sharapova, E A Romanova, L P Sashchenko & D V Yashin. Tag7 (PGLYRP1) Can Induce an Emergence of the CD3+CD4+CD25+CD127+ Cells with Antitumor Activity. *Journal of Immunology Research* **2018**, 4501273-9 (2018).

15. Sharapova, T. N. *et al.* Innate Immunity Protein Tag7 Induces 3 Distinct Populations of Cytotoxic Cells That Use Different Mechanisms to Exhibit Their Antitumor Activity on Human Leukocyte Antigen-Deficient Cancer Cells. *J Innate Immun* **9**, 598-608 (2017).

16. Saha, S. *et al*. Peptidoglycan Recognition Proteins Protect Mice from Experimental Colitis by Promoting Normal Gut Flora and Preventing Induction of Interferon-γ. *Cell Host & Microbe* **8**, 147-162 (2010).

17. Jana, N. K., Gray, L. R. & Shugars, D. C. Human immunodeficiency virus type 1 stimulates the expression and production of secretory leukocyte protease inhibitor (SLPI) in oral epithelial cells: a role for SLPI in innate mucosal immunity. *J. Virol.* **79**, 6432-6440 (2005).

18. Ashcroft, G. S. *et al*. Secretory leukocyte protease inhibitor mediates non-redundant functions necessary for normal wound healing. *Nat. Med.* **6**, 1147-1153 (2000).

19. Araujo, F. M. *et al*. Increased expression of ID2, PRELP and SMOC2 genes in patients with endometriosis. *Braz. J. Med. Biol. Res.* **50**, e5782 (2017).

20. Wieczorek, A. & Balwierz, W. The Role of Id2 Protein in Neuroblatoma in Children. *Pathol. Oncol. Res.* **21**, 999-1004 (2015).

21. Duffield, G. E. *et al*. A role for Id2 in regulating photic entrainment of the mammalian circadian system. *Curr. Biol.* **19**, 297-304 (2009).

22. Li, J. *et al*. Id2 Collaborates with Id3 To Suppress Invariant NKT and Innate-like Tumors. *J. Immunol.* **198**, 3136-3148 (2017).

23. Myers, C. T. *et al*. De Novo Mutations in PPP3CA Cause Severe Neurodevelopmental Disease with Seizures. *Am. J. Hum. Genet.* **101**, 516-524 (2017).

24. Molkentin, J. D. *et al*. A calcineurin-dependent transcriptional pathway for cardiac hypertrophy. *Cell* **93**, 215-228 (1998).

25. Zhang, B. W. *et al*. T cell responses in calcineurin A alpha-deficient mice. *J. Exp. Med.* **183**, 413-420 (1996).

26. Wang, W. *et al*. FABP5 correlates with poor prognosis and promotes tumor cell growth and metastasis in cervical cancer. *Tumour Biol.* **37**, 14873-14883 (2016).

27. Ogawa, E. *et al*. Epidermal FABP (FABP5) regulates keratinocyte differentiation by 13(S)-HODE-mediated activation of the NF-κB signaling pathway. *J. Invest. Dermatol.* **131**, 604-612 (2011).

28. Takebayashi, K. *et al*. Circulating SerpinB1 levels and clinical features in patients with type 2 diabetes. *BMJ Open Diabetes Res Care* **4**, e000274 (2016).

29. El Ouaamari, A. *et al*. SerpinB1 Promotes Pancreatic β Cell Proliferation. *Cell Metab.* **23**, 194-205 (2016).

30. Zhang, T. *et al*. DDX39 promotes hepatocellular carcinoma growth and metastasis through activating Wnt/β-catenin pathway. *Cell Death Dis* **9**, 675 (2018).

31. Otake, K. *et al*. Identification of DDX39A as a Potential Biomarker for Unfavorable Neuroblastoma Using a Proteomic Approach. *Pediatr Blood Cancer* **63**, 221-227 (2016).

32. Pan, Y. *et al*. The Critical Role of Rab31 in Cell Proliferation and Apoptosis in Cancer Progression. *Mol. Neurobiol.* **53**, 4431-4437 (2016).

33. Chua, C. E. L. & Tang, B. L. The role of the small GTPase Rab31 in cancer. *J. Cell. Mol. Med.* **19**, 1-10 (2015).

34. Woodruff, P. G. *et al*. Genome-wide profiling identifies epithelial cell genes associated with asthma and with treatment response to corticosteroids. *PNAS* **104**, 15858-15863 (2007).

35. Palafox-Sánchez, C. A. *et al*. A functional Ser(413)/Ser(413) PAI-2 polymorphism is associated with susceptibility and damage index score in systemic lupus erythematosus. *Clin. Appl. Thromb. Hemost.* **15**, 233-238 (2009).

36. Tai, A. W. *et al*. A functional genomic screen identifies cellular cofactors of hepatitis C virus replication. *Cell Host Microbe* **5**, 298-307 (2009).

37. Karlas, A. *et al*. Genome-wide RNAi screen identifies human host factors crucial for influenza virus replication. *Nature* **463**, 818-822 (2010).

38. Schroder, W. A., Major, L. & Suhrbier, A. The role of SerpinB2 in immunity. *Crit. Rev. Immunol.* **31**, 15-30 (2011).

39. Schroder, W. A. *et al*. A physiological function of inflammation-associated SerpinB2 is regulation of adaptive immunity. *J. Immunol.* **184**, 2663-2670 (2010).

40. Ather, J. L. & Poynter, M. E. Serum amyloid A3 is required for normal weight and immunometabolic function in mice. *PLoS ONE* **13**, e0192352 (2018).

41. Mancardi, D. A. *et al*. Cutting Edge: The murine high-affinity IgG receptor FcγRIV is sufficient for autoantibody-induced arthritis. *J. Immunol.* **186**, 1899-1903 (2011).

42. Nimmerjahn, F., Bruhns, P., Horiuchi, K. & Ravetch, J. V. FcgammaRIV: a novel FcR with distinct IgG subclass specificity. *Immunity* **23**, 41-51 (2005).

43. Falk Nimmerjahn *et al*. FcγRIV deletion reveals its central role for IgG2a and IgG2b activity in vivo. *Proceedings of the National Academy of Sciences of the United States of America* **107**, 19396-19401 (2010).

44. O'Donnell, L. C., Druhan, L. J. & Avalos, B. R. Molecular characterization and expression analysis of leucine-rich alpha2-glycoprotein, a novel marker of granulocytic differentiation. *J. Leukoc. Biol.* **72**, 478-485 (2002).

45. Wang, X. *et al*. LRG1 promotes angiogenesis by modulating endothelial TGF-β signalling. *Nature* **499**, 306-311 (2013).

46. Ge, C., Li, Q., Wang, L. & Xu, X. The role of axon guidance factor semaphorin 6B in the invasion and metastasis of gastric cancer. *J. Int. Med. Res.* **41**, 284-292 (2013).

47. D'Apice, L. *et al*. Analysis of SEMA6B gene expression in breast cancer: identification of a new isoform. *Biochim. Biophys. Acta* **1830**, 4543-4553 (2013).

48. Slade, C. *et al*. Deficiency in complement factor B. *N. Engl. J. Med.* **369**, 1667-1669 (2013).

49. Gold, B. *et al*. Variation in factor B (BF) and complement component 2 (C2) genes is associated with age-related macular degeneration. *Nat. Genet.* **38**, 458-462 (2006).

50. Fojo, S. S. & Brewer, H. B. Hypertriglyceridaemia due to genetic defects in lipoprotein lipase and apolipoprotein C-II. *J. Intern. Med.* **231**, 669-677 (1992).

51. Wolska, A. *et al*. Apolipoprotein C-II: New findings related to genetics, biochemistry, and role in triglyceride metabolism. *Atherosclerosis* **267**, 49-60 (2017).

52. Shachter, N. S. *et al*. Overexpression of apolipoprotein CII causes hypertriglyceridemia in transgenic mice. *J. Clin. Invest.* **93**, 1683-1690 (1994).

53. de Knijff, P., van den Maagdenberg, A. M., Frants, R. R. & Havekes, L. M. Genetic heterogeneity of apolipoprotein E and its influence on plasma lipid and lipoprotein levels. *Hum. Mutat.* **4**, 178-194 (1994).

54. Frikke-Schmidt, R., Sing, C. F., Nordestgaard, B. G., Steffensen, R. & Tybjaerg-Hansen, A. Subsets of SNPs define rare genotype classes that predict ischemic heart disease. *Hum. Genet.* **120**, 865-877 (2007).

55. Mahley, R. Apolipoprotein E: from cardiovascular disease to neurodegenerative disorders. *J Mol Med* **94**, 739-746 (2016).

56. Liao, F., Yoon, H. & Kim, J. Apolipoprotein E metabolism and functions in brain and its role in Alzheimer's disease. *Curr. Opin. Lipidol.* **28**, 60-67 (2017).

57. Han, Z., Huang, H., Gao, Y. & Huang, Q. Functional annotation of Alzheimer's disease associated loci revealed by GWASs. *PLoS ONE* **12**, e0179677 (2017).

58. Zhang, S. H., Reddick, R. L., Piedrahita, J. A. & Maeda, N. Spontaneous hypercholesterolemia and arterial lesions in mice lacking apolipoprotein E. *Science* **258**, 468-471 (1992).

59. Mar, K. B. *et al*. LY6E mediates an evolutionarily conserved enhancement of virus infection by targeting a late entry step. *Nat Commun* **9**, 3603 (2018).

60. Yu, J., Liang, C. & Liu, S. Interferon-inducible LY6E Protein Promotes HIV-1 Infection. *J. Biol. Chem.* **292**, 4674-4685 (2017).

61. Pendergraft, W. F. *et al*. Autoimmunity is triggered by cPR-3(105-201), a protein complementary to human autoantigen proteinase-3. *Nat. Med.* **10**, 72-79 (2004).

62. Martin, K. R. & Witko-Sarsat, V. Proteinase 3: the odd one out that became an autoantigen. *J. Leukoc. Biol.* **102**, 689-698 (2017).

63. Loison, F. *et al*. Proteinase 3-dependent caspase-3 cleavage modulates neutrophil death and inflammation. *J. Clin. Invest.* **124**, 4445-4458 (2014).

64. Lintner, K. E. *et al*. Early Components of the Complement Classical Activation Pathway in Human Systemic Autoimmune Diseases. *Front Immunol* **7**, 36 (2016).

65. Wessels, M. R. *et al*. Studies of group B streptococcal infection in mice deficient in complement component C3 or C4 demonstrate an essential role for complement in both innate and acquired immunity. *Proc. Natl. Acad. Sci. U. S. A.* **92**, 11490-11494 (1995).

66. Fischer, M. B. *et al*. Regulation of the B cell response to T-dependent antigens by classical pathway complement. *J. Immunol.* **157**, 549-556 (1996).

67. Garrido-Sanchez, L. *et al*. Munc18c in adipose tissue is downregulated in obesity and is associated with insulin. *PLoS ONE* **8**, e63937 (2013).

68. Ramalingam, Latha|Yoder, Stephanie M.|Oh, Eunjin|Thurmond, Debbie C. Munc18c: a controversial regulator of peripheral insulin action. *Trends in Endocrinology & Metabolism* **25**, 601-608 (2014).

69. Oh, E., Spurlin, B. A., Pessin, J. E. & Thurmond, D. C. Munc18c heterozygous knockout mice display increased susceptibility for severe glucose intolerance. *Diabetes* **54**, 638-647 (2005).

70. Ma, J. *et al*. MS4A6A genotypes are associated with the atrophy rates of Alzheimer's disease related brain structures. *Oncotarget* **7**, 58779-58788 (2016).

71. Yano, H. *et al*. The significance of TIMD4 expression in clear cell renal cell carcinoma. *Med Mol Morphol* **50**, 220-226 (2017).

72. Xu, L. *et al*. Tim-4 Inhibits NO Generation by Murine Macrophages: e0124771. *PLoS ONE* **10** (2015).

73. Tsuchiya, N. & Kyogoku, C. Role of Fc gamma receptor IIb polymorphism in the genetic background of systemic lupus erythematosus: insights from Asia. *Autoimmunity* **38**, 347-352 (2005).

74. Bosch, R. *et al*. FcγRIIb expression in early stage chronic lymphocytic leukemia. *Leuk. Lymphoma* **58**, 2642-2648 (2017).

75. Bolland, S. & Ravetch, J. V. Spontaneous autoimmune disease in Fc(gamma)RIIB-deficient mice results from strain-specific epistasis. *Immunity* **13**, 277-285 (2000).

76. Bolland, S., Yim, Y., Tus, K., Wakeland, E. K. & Ravetch, J. V. Genetic modifiers of systemic lupus erythematosus in FcgammaRIIB(-/-) mice. *J. Exp. Med.* **195**, 1167-1174 (2002).

77. Ekoue, D. N. *et al*. Selenium levels in human breast carcinoma tissue are associated with a common polymorphism in the gene for SELENOP (Selenoprotein P). *J Trace Elem Med Biol* **39**, 227-233 (2017).

78. Pellatt, A. J. *et al*. SEPP1 influences breast cancer risk among women with greater native american ancestry: the breast cancer health disparities study. *PLoS ONE* **8**, e80554 (2013).

79. Hill, K. E., Zhou, J., McMahan, W. J., Motley, A. K. & Burk, R. F. Neurological dysfunction occurs in mice with targeted deletion of the selenoprotein P gene. *J. Nutr.* **134**, 157-161 (2004).

80. Caito, S. W. *et al*. Progression of neurodegeneration and morphologic changes in the brains of juvenile mice with selenoprotein P deleted. *Brain Res.* **1398**, 1-12 (2011).

81. Jiang, X. The effect of phospholipid transfer protein on lipoprotein metabolism and atherosclerosis. *Front. Biosci.* **7**, 1634 (2002).

82. Qin, S., Song, G. & Yu, Y. Phospholipid transfer protein in diabetes, metabolic syndrome and obesity. *Cardiovasc Hematol Disord Drug Targets* **14**, 149-153 (2014).

83. Jiang, X. C. *et al*. Targeted mutation of plasma phospholipid transfer protein gene markedly reduces high-density lipoprotein levels. *J. Clin. Invest.* **103**, 907-914 (1999).

84. Liu, J. & Zhu, H. TMEM106A inhibits cell proliferation, migration, and induces apoptosis of lung cancer cells. *J. Cell. Biochem.* (2018).

85. Wu, C. *et al*. TMEM106a is a Novel Tumor Suppressor in Human Renal Cancer. *Kidney Blood Press. Res.* **42**, 853-864 (2017).

86. Dai, H., Xu, D., Su, J., Jang, J. & Chen, Y. Transmembrane protein 106a activates mouse peritoneal macrophages via the MAPK and NF-κB signaling pathways. *Sci Rep* **5**, 12461 (2015).

87. Gao, D. *et al*. Methylation of TMEM176A is an independent prognostic marker and is involved in human colorectal cancer development. *Epigenetics* **12**, 575-583 (2017).

88. Li, H. *et al*. Epigenetic silencing of TMEM176A activates ERK signaling in human hepatocellular carcinoma. *Clin Epigenetics* **10**, 137 (2018).

89. Sun, C. *et al*. Expression of annexin A5 in serum and tumor tissue of patients with colon cancer and its clinical significance. *World J. Gastroenterol.* **23**, 7168-7173 (2017).

90. Rogenhofer, N. *et al*. Assessment of M2/ANXA5 haplotype as a risk factor in couples with placenta-mediated pregnancy complications. *J. Assist. Reprod. Genet.* **35**, 157-163 (2018).

91. Aranda, F. *et al*. Maternal carriers of the ANXA5 M2 haplotype are exposed to a greater risk for placenta-mediated pregnancy complications. *J. Assist. Reprod. Genet.* **35**, 921-928 (2018).

92. Scarpellino, L. *et al*. Interactions of Ly49 family receptors with MHC class I ligands in trans and cis. *J. Immunol.* **178**, 1277-1284 (2007).

93. Cai, Y. *et al*. Associations of TF Gene Polymorphisms with the Risk of Ischemic Stroke. *J. Mol. Neurosci.* **65**, 359-366 (2018).

94. Si, Q. *et al*. Plasma transferrin level correlates with the tremor-dominant phenotype of Parkinson's disease. *Neurosci. Lett.* **684**, 42-46 (2018).

95. Rodero, M. P. *et al*. Type I interferon-mediated autoinflammation due to DNase II deficiency. *Nat Commun* **8**, 2176 (2017).

96. Kimura-Kataoka, K. *et al*. Seven nonsynonymous SNPs in the gene encoding human deoxyribonuclease II may serve as a functional SNP potentially implicated in autoimmune dysfunction. *Electrophoresis* **34**, 3361-3369 (2013).

97. Kawane, K. *et al*. Chronic polyarthritis caused by mammalian DNA that escapes from degradation in macrophages. *Nature* **443**, 998-1002 (2006).

98. Wang, S. *et al*. Association between bone marrow stromal cell antigen 1 gene polymorphisms and the susceptibility to Parkinson's disease: a meta-analysis. *Neurosci. Lett.* **599**, 120-124 (2015).

99. Lopatina, O. *et al*. Anxiety- and depression-like behavior in mice lacking the CD157/BST1 gene, a risk factor for Parkinson's disease. *Front Behav Neurosci* **8**, 133 (2014).

100. Künzel, U. *et al*. FRMD8 promotes inflammatory and growth factor signalling by stabilising the iRhom/ADAM17 sheddase complex. *Elife* **7** (2018).

101. Wu, Y., Huang, C., Meng, X. & Li, J. Long Noncoding RNA MALAT1: Insights into its Biogenesis and Implications in Human Disease. *Curr. Pharm. Des.* **21**, 5017-5028 (2015).

102. Battelli, M. G., Polito, L., Bortolotti, M. & Bolognesi, A. Xanthine oxidoreductase in cancer: more than a differentiation marker. *Cancer Med* **5**, 546-557 (2016).

103. Liu, H. *et al*. Genetic variants in XDH are associated with prognosis for gastric cancer in a Chinese population. *Gene* **663**, 196-202 (2018).

104. Piret, S. E. *et al*. A mouse model of early-onset renal failure due to a xanthine dehydrogenase nonsense mutation. *PLoS ONE* **7**, e45217 (2012).
