# Supplementary material for: ImmGen report: sexual dimorphism in the immune system transcriptome
Source: Nat Commun. 2019 Sep 20;10:4295. doi: 10.1038/s41467-019-12348-6 (PMC6754408; doi:10.1038/s41467-019-12348-6)
Supplement: Supplementary file 3 — Reporting Summary [file 41467_2019_12348_MOESM3_ESM.pdf]

nature research

Corresponding author(s): Tal Shay

Last updated by author(s): 25-08-2019

Reporting Summary

Nature Research wishes to improve the reproducibility of the work that we publish. This form provides structure for consistency and transparency in reporting. For further information on Nature Research policies, see Authors & Referees and the Editorial Policy Checklist.

Statistics

For all statistical analyses, confirm that the following items are present in the figure legend, table legend, main text, or Methods section.

n/a	Confirmed	
☐	☒	The exact sample size (*n*) for each experimental group/condition, given as a discrete number and unit of measurement
☐	☒	A statement on whether measurements were taken from distinct samples or whether the same sample was measured repeatedly
☐	☒	The statistical test(s) used AND whether they are one- or two-sided<br>*Only common tests should be described solely by name; describe more complex techniques in the Methods section.*
☐	☒	A description of all covariates tested
☐	☒	A description of any assumptions or corrections, such as tests of normality and adjustment for multiple comparisons
☐	☒	A full description of the statistical parameters including central tendency (e.g. means) or other basic estimates (e.g. regression coefficient) AND variation (e.g. standard deviation) or associated estimates of uncertainty (e.g. confidence intervals)
☒	☐	For null hypothesis testing, the test statistic (e.g. *F*, *t*, *r*) with confidence intervals, effect sizes, degrees of freedom and *P* value noted<br>*Give P values as exact values whenever suitable.*
☒	☐	For Bayesian analysis, information on the choice of priors and Markov chain Monte Carlo settings
☒	☐	For hierarchical and complex designs, identification of the appropriate level for tests and full reporting of outcomes
☒	☐	Estimates of effect sizes (e.g. Cohen's *d*, Pearson's *r*), indicating how they were calculated

Our web collection on statistics for biologists contains articles on many of the points above.

Software and code

Policy information about availability of computer code

Data collection	*Provide a description of all commercial, open source and custom code used to collect the data in this study, specifying the version used OR state that no software was used.*
Data analysis	The code used for the analysis of differential OCRs was deposit in GitHub and publicly available at DOI: 10.5281/zenodo.3377594

For manuscripts utilizing custom algorithms or software that are central to the research but not yet described in published literature, software must be made available to editors/reviewers. We strongly encourage code deposition in a community repository (e.g. GitHub). See the Nature Research guidelines for submitting code & software for further information.

Data

Policy information about availability of data

All manuscripts must include a data availability statement. This statement should provide the following information, where applicable:

- Accession codes, unique identifiers, or web links for publicly available datasets
- A list of figures that have associated raw data
- A description of any restrictions on data availability

All RNA-seq data sets generated in this manuscript have been deposited in the GEO under accession number GSE124829 [https://www.ncbi.nlm.nih.gov/geo/query/acc.cgi?acc=GSE124829]. Samples of dataset C are also part of GSE122108 [https://www.ncbi.nlm.nih.gov/geo/query/acc.cgi?acc=GSE122108]. ATAC-seq samples are part of GSE100738 [https://www.ncbi.nlm.nih.gov/geo/query/acc.cgi?acc=GSE100738]. Human ImmVar data accession number is GSE56035 [https://www.ncbi.nlm.nih.gov/geo/query/acc.cgi?acc=GSE56035]. The source data underlying all Figures and Supplementary Figures are provided as a Source Data file. All other data are included in the supplemental information or available from the authors upon reasonable requests.

Field-specific reporting

Please select the one below that is the best fit for your research. If you are not sure, read the appropriate sections before making your selection.

☒ Life sciences ☐ Behavioural & social sciences ☐ Ecological, evolutionary & environmental sciences

For a reference copy of the document with all sections, see nature.com/documents/nr-reporting-summary-flat.pdf

Life sciences study design

All studies must disclose on these points even when the disclosure is negative.

Sample size

Data was collected in four independent experiments for a total of 92 female samples and 91 male samples. The four datasets were composed as follows (Fig. 1a, Supplementary Table 1): Dataset A ("11-cell-set" ages) included 2-4 repeats for 3 different ages (young - 2 months; adult - 6 months; and old - 17/20 months in females/males, respectively) for 11 unstimulated immune cell types from males and females (66 samples in total). Dataset B ("11-cell-set" NVE-IFN) included 3 repeats for 6 weeks old male and female of 11 unstimulated (NVE) immune cell types from males and females (66 samples in total) and 24 samples of 3 immune cell types (B, GN, MF) after stimulation by type-1 interferon (IFN), with triplicates for 6 weeks old male and female of 10K IFN for each cell type and also 1K IFN for B cells (90 samples in total). Dataset C (tissue MFs) comprised 2-3 replicates for 6 weeks (peritoneal cavity and spleen) and 8 weeks (CNS) old male and female of unstimulated MFs originating from the peritoneal cavity (PC), the spleen (Sp) or the central nervous system (CNS). While PC and Sp originated from the same pool of mice, CNS samples were produced from independent mice pool (15 samples in total). Dataset D (ATAC-seq matched mRNA) included 2 repeats of 3 (of 11 cell set) cell types, namely, MF, B cells and T4 cells, of ultra-low input (ULI) RNA-seq of the ATAC-seq samples.

Data exclusions

Samples with less than one million mapped and paired reads were not included in the downstream analysis. Of the 196 samples produced, 13 were discarded due to low quality (4 from dataset A, 6 from IFN-induced male and female T4 cells from dataset B, and 3 from the tissue MF dataset C)

Replication

The 4 independent datasets were used to replicate findings when relevant

Randomization

All B6 mice. No randomization of experimental group assignment.

Blinding

Blinding impossible for male and female.

Reporting for specific materials, systems and methods

We require information from authors about some types of materials, experimental systems and methods used in many studies. Here, indicate whether each material, system or method listed is relevant to your study. If you are not sure if a list item applies to your research, read the appropriate section before selecting a response.

Materials & experimental systems

n/a	Involved in the study	
☐	☒	Antibodies
☒	☐	Eukaryotic cell lines
☒	☐	Palaeontology
☐	☒	Animals and other organisms
☒	☐	Human research participants
☒	☐	Clinical data

Methods

n/a	Involved in the study	
☒	☐	ChIP-seq
☐	☒	Flow cytometry
☒	☐	MRI-based neuroimaging

Antibodies

Antibodies used

eBioscience 48-0193,
53-1021,12-0431,25-4801,17-0051,48-5961,11-5890,12-0621,25-0193,17-0081,47-0042,48-5961,48-0452-82,11-5321,25-4801,
56-0114-82,12-1351-82,17-5931-82,48-5961,11-0042,12-5941,46-5711,17-0251

Validation

Describe the validation of each primary antibody for the species and application, noting any validation statements on the manufacturer's website, relevant citations, antibody profiles in online databases, or data provided in the manuscript.

Animals and other organisms

Policy information about studies involving animals; ARRIVE guidelines recommended for reporting animal research

Laboratory animals

Male and female C56Bl/6J inbred mice. Ages - 6 weeks, 2, 6, 17 and 20 months

Wild animals	The study did not involve wild animals.
Field-collected samples	The study did not involve samples from the fields.
Ethics oversight	Harvard Medical School, under Institutional Animal Care and Use Committee protocol IS1257

Note that full information on the approval of the study protocol must also be provided in the manuscript.

Flow Cytometry

Plots

Confirm that:

- [x] The axis labels state the marker and fluorochrome used (e.g. CD4-FITC).
- [ ] The axis scales are clearly visible. Include numbers along axes only for bottom left plot of group (a 'group' is an analysis of identical markers).
- [x] All plots are contour plots with outliers or pseudocolor plots.
- [ ] A numerical value for number of cells or percentage (with statistics) is provided.

Methodology

Sample preparation	Standard splenocyte sorts. https://www.immgen.org/Protocols/ImmGen%20Cell%20prep%20and%20sorting%20SOP.pdf
Instrument	BD FACSAriaTM Cell Sorter IIu is a 561 laser flow cytometer with 15 fluorescence detectors
Software	Flow cytometry data was collected using BD FACSDIVATM Software and analyzed using FlowJo v10
Cell population abundance	In most cases, a carefully selected dump channel was used to exclude certain populations. All populations were then double sorted for a final of 1000 cells per sample to achieve >99% sort purity
Gating strategy	For all 13 populations, we used FSC-H/FSC-A and FSC-A/SSC-A gates to differentiate between singlets/doublets and viable cells/ cell debris. The live cells were then identified using PI, which excluded the positive dead cells. The peritoneal B1aB cells and the macrophages were identified as F480+ICAM2+ and CD19+CD5+ respectively. The splenic CD4 T cells and CD8 T cells were identified as TCRB+CD4+ and TCRB+CD8+ respectively. B cells were identified as CD19+IgM+ and macrophage as F480+MHCIIint. After excluding B and T cells via a dump channel, dendritic cells and neutrophils were identified as CD11c+FLT3+ and Ly-6G+ respectively. In addition, in one panel, a stringent dump channel was used to exclude CD8 T, B, erythroid and myeloid cells, leading to the identification of NK and TCRgd as NK1.1+TCRb- and TCRgd+TCRb-. Tregs and NKT were then identified as TCRb +CD4+CD25+ and NK1.1+TCRb+. Finally cerebral macrophages were identified as CD45intCD11b+

- [x] Tick this box to confirm that a figure exemplifying the gating strategy is provided in the Supplementary Information.
