# Supplementary material for: ImmGen report: sexual dimorphism in the immune system transcriptome
Source: Nat Commun. 2019 Sep 20;10:4295. doi: 10.1038/s41467-019-12348-6 (PMC6754408; doi:10.1038/s41467-019-12348-6)
Supplement: Supplementary file 4 — Description of Additional Supplementary Files [file 41467_2019_12348_MOESM4_ESM.pdf]

Description of Additional Supplementary Files

File Name: Supplementary Data 1

Description:

Details of the RNA-sequencing datasets used in this study. Including dataset name, cell type, tissue of origin and number of replicates in the first sheet. In the second sheet the sorting panels are detailed with sorting markers and antibodies used, their color, dilution and catalogue number. See Supplementary Figure 8 for the panels figures.

File Name: Supplementary Data 2

Description:

Up-regulated genes in macrophages from IFN stimulated mice compared to unstimulated mice. For each gene, expression level and statistical parameters are given. Macrophages from females are in the first sheet, and macrophages from males in the second sheet.

File Name: Supplementary Data 3

Description:

Two-Way ANOVA results presenting the differentially expressed genes (as well as Fold change and P values) in male and female macrophages from three different tissues. Sex effect in the first sheet and sex*tissue interaction effect in the second sheet.

File Name: Supplementary Data 4

Description:

Differentially expressed genes between male and female human (ImmVar results) immune cell types, together with the murine male-female fold change of the orthologous genes. CD14 in the first sheet and CD4 T cells in the second sheet.
